# Low‐intensity transcranial magnetic stimulation promotes the survival and maturation of newborn oligodendrocytes in the adult mouse brain

**DOI:** 10.1002/glia.23620

**Published:** 2019-04-16

**Authors:** Carlie L. Cullen, Matteo Senesi, Alexander D. Tang, Mackenzie T. Clutterbuck, Loic Auderset, Megan E. O'Rourke, Jennifer Rodger, Kaylene M. Young

**Affiliations:** ^1^ Menzies Institute for Medical Research, University of Tasmania Hobart Tasmania Australia; ^2^ Experimental and Regenerative Neurosciences, School of Biological Sciences University of Western Australia Perth Western Australia Australia; ^3^ Brain Plasticity Lab Perron Institute for Neurological and Translational Science Perth Western Australia Australia

**Keywords:** adaptive myelination, cortex, internode, myelin, oligodendrocyte survival, oligodendrogenesis, transcranial magnetic stimulation

## Abstract

Neuronal activity is a potent extrinsic regulator of oligodendrocyte generation and central nervous system myelination. Clinically, repetitive transcranial magnetic stimulation (rTMS) is delivered to noninvasively modulate neuronal activity; however, the ability of rTMS to facilitate adaptive myelination has not been explored. By performing cre‐lox lineage tracing, to follow the fate of oligodendrocyte progenitor cells in the adult mouse brain, we determined that low intensity rTMS (LI‐rTMS), administered as an intermittent theta burst stimulation, but not as a continuous theta burst or 10 Hz stimulation, increased the number of newborn oligodendrocytes in the adult mouse cortex. LI‐rTMS did not alter oligodendrogenesis per se, but instead increased cell survival and enhanced myelination. These data suggest that LI‐rTMS can be used to noninvasively promote myelin addition to the brain, which has potential implications for the treatment of demyelinating diseases such as multiple sclerosis.

## Main Points

- The impact that low intensity repetitive transcranial magnetic stimulation (LI‐rTMS) has on cells of the oligodendrocyte lineage is stimulation frequency and cell‐stage specific.
- LI‐rTMS does not affect oligodendrocyte progenitor cell proliferation or density.
- LI‐rTMS, delivered in an intermittent theta burst pattern, promotes the survival of premyelinating oligodendrocytes and enhances myelin internode extension by newly myelinating oligodendrocytes.

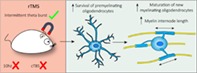

## 1 INTRODUCTION

Myelinating oligodendrocytes are added to the central nervous system (CNS) throughout life, generated from immature, proliferative oligodendrocyte progenitor cells (OPCs), also known as NG2‐glia (reviewed by Pepper, Pitman, Cullen, & Young, 2018). In development and adulthood, myelination is regulated by many intrinsic and extrinsic factors, with neuronal activity being a major extrinsic regulator of adaptive myelination (reviewed by Bechler, Swire, & Ffrench‐Constant, 2017), influencing OPC proliferation (Barres & Raff, 1993; Gibson et al., 2014), oligodendrogenesis (Gibson et al., 2014; Li, Brus‐Ramer, Martin, & McDonald, 2010), oligodendrocyte survival (Barres, Jacobson, Schmid, Sendtner, & Raff, 1993; Kougioumtzidou et al., 2017), myelin sheath stabilization (Hines, Ravanelli, Schwindt, Scott, & Appel, 2015), the number of internodes supported per oligodendrocyte (Mensch et al., 2015), and myelin sheath thickness (Gibson et al., 2014). The ability of neuronal activity to promote oligodendrogenesis and myelination also makes it an interesting therapeutic target for myelin repair.

Repetitive transcranial magnetic stimulation (rTMS) is a safe and noninvasive form of neural stimulation that applies focal magnetic fields to generate electric currents in the brain (Barker, Freeston, Jalinous, Merton, & Morton, 1985) and can increase or decrease neuronal firing depending on the intensity, frequency, and pattern of stimulation (Hoppenrath, Hartig, & Funke, 2016; Müller‐Dahlhaus & Vlachos, 2013; Tang, Thickbroom, & Rodger, 2017). rTMS exerts this effect on neuronal activity by modulating the activity of gamma‐aminobutyric acid (GABA)‐ and glutamate‐releasing neurons (Croarkin et al., 2016; Hoppenrath & Funke, 2013; Lenz et al., 2015; Lenz et al., 2016; Vlachos et al., 2012), increasing intracellular calcium levels (Grehl et al., 2015) and promoting the release of growth factors such as brain‐derived neurotrophic factor (BDNF) (Castillo‐Padilla & Funke, 2016; Makowiecki, Harvey, Sherrard, & Rodger, 2014; Müller, Toschi, Kresse, Post, & Keck, 2000; Zhang, Xing, Wang, Tao, & Cheng, 2015)—all of which are key regulators of oligodendrogenesis and adaptive myelination (Gautier et al., 2015; Hamilton et al., 2017; Pitman & Young, 2016; Wong, Xiao, Kemper, Kilpatrick, & Murray, 2013; Xiao et al., 2010). For these reasons, rTMS has the potential to influence adaptive myelination, and ultimately find application in the repair of demyelinated lesions in the CNS of people with multiple sclerosis (MS).

The small number of clinical trials that have explored the benefit of rTMS for people with MS have reported a reduction in fatigue (Gaede et al., 2018; Mori et al., 2011) and muscle spasticity (Mori et al., 2010; Mori et al., 2011; Nielsen, Sinkjaer, & Jakobsen, 1996), as well as improved functional connectivity and working memory performance (Hulst et al., 2017); however, the effect that rTMS has on oligodendrocyte and myelin parameters has not been examined in this context. In rat models of demyelination, lesion size is reduced when repetitive magnetic stimulation is delivered from the day of lysolecithin (Sherafat et al., 2012) or ethidium bromide (Fang, Li, Xiong, Huang, & Huang, 2010) injection into the corpus callosum and spinal cord, respectively. Furthermore, magnetic brain stimulation can reduce disability when delivered at the onset of experimental autoimmune encephalomyelitis (Medina‐Fernández et al., 2017). While these studies suggest that magnetic stimulation can influence oligodendrocyte or myelin loss and/or replacement, the capacity for rTMS to increase oligodendrocyte generation or myelination, even in the healthy brain, has not been explored (Cullen & Young, 2016).

Herein, we demonstrate that the delivery of low intensity rTMS (LI‐rTMS; 120 mT) in an intermittent theta‐burst pattern (iTBS), can increase the number of newborn or newly differentiated oligodendrocytes that survive and mature in the cortex, and enhance myelin internode extension by differentiating oligodendrocytes, effectively promoting myelination of the healthy CNS.

## 2 MATERIALS AND METHODS

### 2.1 Animal housing

Male and female mice were group‐housed, separately, in individually ventilated cages (12 hr light cycle, 21°C) with ad libitum access to food and water. Mice were randomly assigned to each treatment, but care was taken to ensure littermates were represented across treatment groups. All animal experiments were approved by the University of Tasmania Animal Ethics Committee and carried out in accordance with the Australian code of practice for the care and use of animals in science.

### 2.2 Transgenic lineage tracing

For the lineage tracing of OPCs, heterozygous *Pdgfrα‐CreER* ^*T2*^ transgenic mice (Rivers et al., 2008) were crossed with either homozygous *Rosa26‐YFP* (Srinivas et al., 2001) or heterozygous *Tau‐lox‐STOP‐lox‐mGFP‐IRES‐NLS‐LacZ‐pA* (*Tau‐mGFP*; Hippenmeyer et al., 2005) cre‐sensitive reporter mice to generate double heterozygous offspring. Expression of *Cre* recombinase and *Rosa26‐YFP* was confirmed by polymerase chain reaction (PCR) as described by Rivers et al. (2008) and expression of membrane tethered *GFP* (*GFP*) as described by Young et al. (2013). In brief, genomic DNA was extracted from ear biopsies by ethanol precipitation and PCR performed using 50–100 ng of gDNA with the following primer combinations: Rosa26 wildtype 5′ AAAGT CGCTC TGAGT TGTTAT, Rosa26 wildtype 3′ GGAGC GGGAG AAATG GATATG and Rosa26 YFP 5′ GCGAA GAGTT TGTCC TCAACC; Cre 5′ CAGGT CTCAG GAGCT ATGTC CAATT TACTG ACCGTA and Cre 3′ GGTGT TATAAG CAATCC CCAGAA, or GFP 5′ CCCTG AAGTTC ATCTG CACCAC and GFP 3′ TTCTC GTTGG GGTCT TTGCTC.

To activate Cre‐recombinase and drive expression of the fluorescent reporters, Tamoxifen was dissolved in corn oil (40 mg/mL) by sonication at 21°C for 2 hr and administered to adult mice (P83) by oral gavage at a dose of 300 mg tamoxifen/kg body weight daily for 4 days.

### 2.3 LI‐rTMS

LI‐rTMS was delivered as 600 pulses of 10 Hz (60 s), iTBS (192 s), or cTBS (40 s) (Figure S1) using a custom made 120 mT circular coil designed for rodent stimulation (8 mm outer diameter, iron core; Tang, Lowe, et al., 2016). Stimulation parameters were controlled by a waveform generator (Agilent Technologies, Santa Clara, CA) connected to a bipolar voltage programmable power supply (KEPCO BOP 100‐4 M, TMG test equipment). Experiments were conducted at 100% maximum power output (100 V) using custom monophasic waveforms (400 μs rise time; Agilent Benchlink Waveform Builder; Agilent Technologies, Santa Clara, CA, USA). Mice were restrained using plastic body‐contour shape restraint cones (0.5 mm thick; Abel Scientific). The coil was manually held over the midline of the head with the back of the coil positioned in line with the front of the ears (∼Bregma −3.0; Figure S1). For spinal cord stimulation, the coil was held over the spinal column so that the front of the coil was positioned over the T13 vertebrae (see Figure S2), which was identified by palpating the location of the 13th rib. Sham mice were positioned under the coil for 192 s (as per iTBS), but no current was passed through the coil. Stimulation was carried out once daily, at the same time, for up to 28 consecutive days. LI‐rTMS did not elicit observable behavior changes in the mice during or immediately after stimulation.

### 2.4 Tissue preparation and immunohistochemistry

Mice were perfusion‐fixed with 4% paraformaldehyde (PFA; Sigma) (wt/vol) in phosphate buffered saline (PBS). Brains were cut into 2 mm‐thick coronal slices using a 1 mm brain matrix (Kent Scientific) before being post‐fixed in 4% (wt/vol) PFA at 21°C for 90 min. Tissue was cryoprotected in 20% sucrose (Sigma) in PBS and snap frozen in OCT (ThermoFisher) for storage at −80°C. 30 μm coronal brain cryosections were collected and processed as floating sections (as per O'Rourke et al., 2016). Primary and secondary antibodies were diluted in PBS blocking solution [0.1% (vol/vol) Triton X‐100 and 10% fetal calf serum in PBS] and applied to cryosections overnight at 4°C. Primary antibodies included goat anti‐PDGFRα (1:200; GeneTex, California), rabbit anti‐OLIG2 (1:400 Millipore) and rat anti‐GFP (1:2000; Nacalai Tesque, Kyoto, Japan). Secondary antibodies were conjugated to AlexaFluor‐488, −568 or − 647 (Invitrogen) and included: donkey anti‐goat (1:1,000), donkey anti‐rabbit (1:1,000), and donkey anti‐rat (1:500). Nuclei were labeled using Hoechst 33342 (1:1,000; Invitrogen).

### 2.5 EdU labeling and detection

5‐Ethynyl‐2′‐deoxyuridine (EdU; Invitrogen) was administered via the drinking water at a concentration of 0.2 mg/mL (as per Clarke et al., 2012; Young et al., 2013) for up to 14 days, from Day 1 of sham‐stimulation or LI‐rTMS (P90). EdU labeling was visualised using the AlexaFluor‐647 Click‐IT EdU kit (Invitrogen). Floating cryosections were incubated for 15 min in 0.5% triton x‐100 (vol/vol) in PBS at 21°C, transferred into the EdU developing cocktail, and incubated in the dark for 45 min, before they were washed in PBS to commence immunohistochemistry.

### 2.6 TUNEL labeling

Terminal deoxynucleotidyl transferase mediated 2′‐deoxyuridine, 5′‐triphosphate nick end labeling (TUNEL) was performed using a fluorescein in situ cell detection kit (Roche) as per the manufacturer's instructions. In brief, floating brain cryosections (30 μm) were collected from mice that had received 14 days of iTBS or sham stimulation, immersion fixed in 4% PFA at ∼21°C for 20 min, washed with PBS and permeabilized in 0.1% triton x‐100/0.1% sodium citrate on ice for 2 min. Sections were then PBS washed, incubated in the TUNEL reaction mixture at 37°C in the dark for 1 hr, washed in PBS and counter‐stained with Hoechst 33342 (1:1,000; Invitrogen) before being transferred to glass slides, allowed to dry and coverslipped with fluorescent mounting medium (DAKO).

### 2.7 Confocal microscopy and cell quantification

Confocal images were collected using an UltraView Nikon Ti Microscope with Volocity Software (Perkin Elmer). For cell number quantification, low magnification (20× objective) images were taken of the cortex, corpus callosum (CC) or spinal cord. Multiple z stack images (2 μm spacing) were collected using standard excitation and emission filters for DAPI, FITC (AlexaFluor‐488), TRITC (AlexaFluor‐568) and CY5 (AlexaFluor‐647) and stitched together to make a composite image of a defined region of interest. The stitched cortical images were manually subdivided into specific regions using Image J (NIH) based on Hoechst nuclear staining and according to the Mouse Brain Atlas (Franklin & Paxinos, 2007). Cell quantification was performed manually using the cell counter plugin for Image J (NIH). For quantification of internode number and length, high magnification confocal images (40× objective) were taken through individual oligodendrocytes (0.5 μm steps) that had a visible cell body, myelinating morphology, and fell within the region of interest. Internodes were manually traced in Image J (NIH). All data acquisition was carried out by an experimenter blind to the treatment group.

### 2.8 Statistical analyses

**Figure 1:**
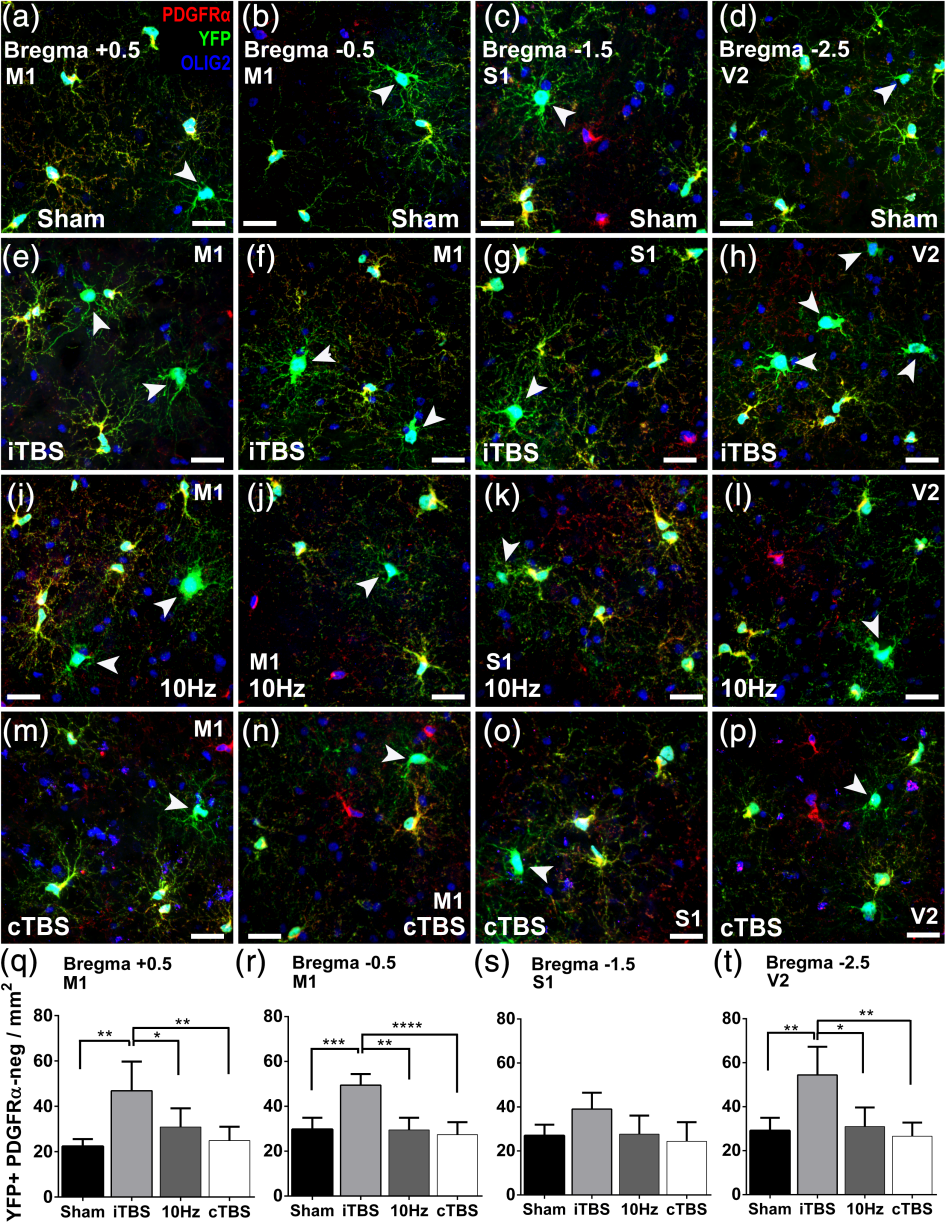
iTBS increases the number of new oligodendrocytes in the adult mouse cortex. (a–p) Representative, compressed confocal image stacks showing PDGFRα (red), YFP (green), and OLIG2 (blue) staining in the primary motor cortex (M1), primary somatosensory cortex (S1), and secondary visual cortex (V2) of adult *Pdgfrα‐CreER* ^*T2*^ *::Rosa26‐YFP* mice that received 14 consecutive days of sham stimulation (a–d), iTBS (e–h), 10 Hz (i–l), or cTBS (m–p) and were analyzed 1 day later. (q–r) Graphical representation of the number of YFP^+^ OLIG2^+^ PDGFRα‐negative new oligodendrocytes within M1, 1 day after 14 days of sham, iTBS, 10 Hz or cTBS treatment (*n* = 5 mice per treatment group) [(q): *Bregma + 0.5, one‐way ANOVA F(3, 16) = 8.65, p = 0.0012;* (r): *Bregma − 0.5, one‐way ANOVA F(3, 16) = 19.14, p = 0.0012]*. (s) Graphical representation of the number of YFP^+^ OLIG2^+^ PDGFRα‐negative new oligodendrocytes within S1, 1 day after 14 days of sham, iTBS, 10 Hz or cTBS treatment *[Bregma − 1.5, n = 5 mice per treatment group, one‐way ANOVA F(3, 16) = 2.99, p = .073]*. (t) Graphical representation of the number of YFP^+^ OLIG2^+^ PDGFRα‐negative new oligodendrocytes within V2, 1 day after 14 days of sham, iTBS, 10 Hz or cTBS treatment *[Bregma − 2.5, n = 5 mice per treatment group, one‐way ANOVA F(3, 16) = 8.53, p = .0026]*. Asterisks denote significant differences identified by Bonferroni post hoc analysis: *p < .05, **p < .01, ***p < .001, and ****p < .0001. Scale bars represent 15 μm. Arrowheads identify YFP^+^ OLIG2^+^ PDGFRα‐negative new oligodendrocytes [Color figure can be viewed at http://wileyonlinelibrary.com]

**Figure 2:**
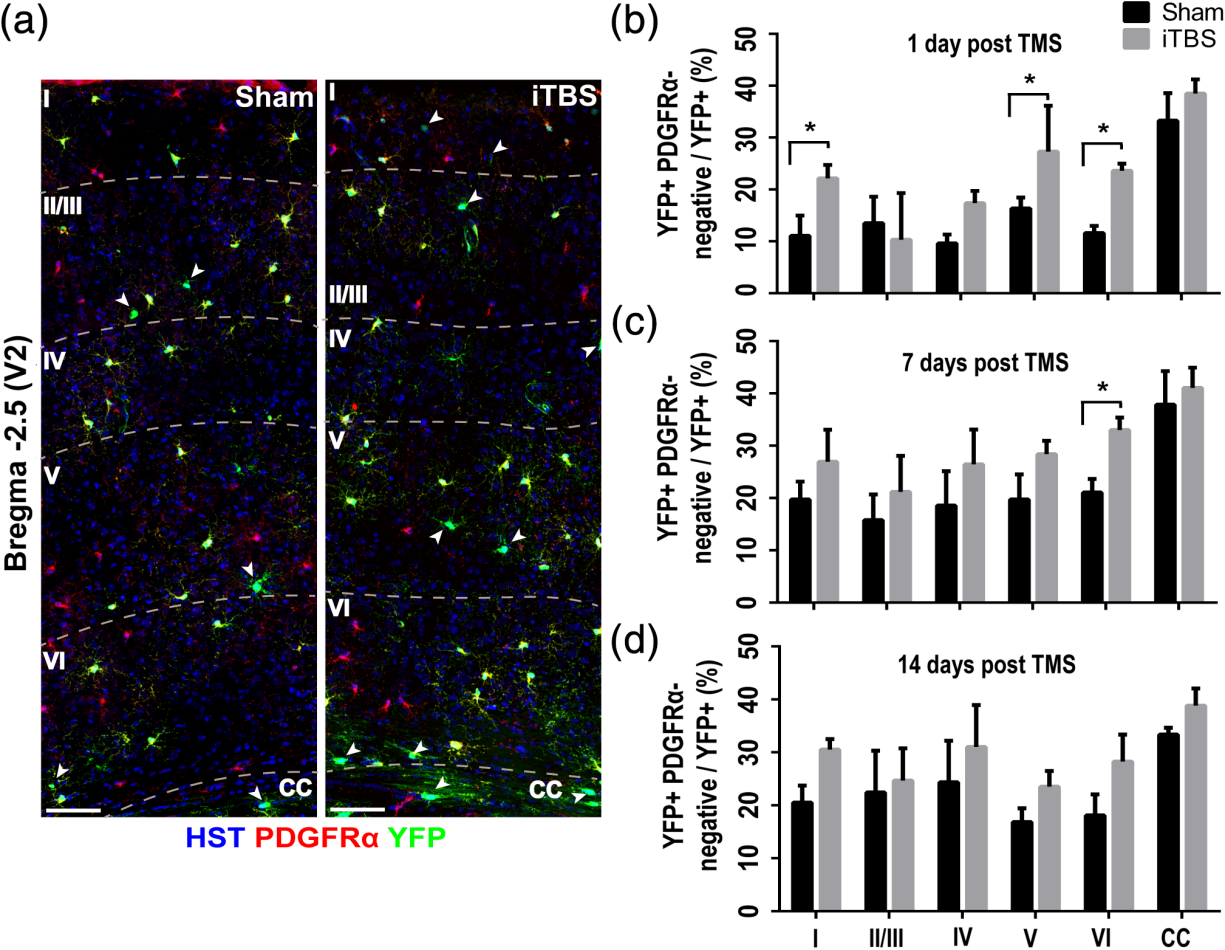
iTBS does not increase new oligodendrocyte number across all cortical layers. (a) Low magnification confocal images of the secondary visual cortex (V2) of *Pdgfrα‐CreER* ^*T2*^ *::Rosa26‐YFP* mice that were perfused 1 day after they received 14 days of sham stimulation (left) or iTBS (right), stained to detect PDGFRα (red), YFP (green) and Hoechst 33342 (HST, blue). (b–d) Graph showing the proportion of YFP^+^ cells that are newly differentiated oligodendrocytes (PDGFRα‐negative, OLIG2^+^) in each layer of V2 and the CC of mice that received 14 days of sham or iTBS treatment and were perfusion fixed for analysis 1 day later *[b: n = 4 mice per treatment, two‐way ANOVA treatment F(1, 36) = 22.26, p < .0001; layer F(5, 36) = 21.08, p < .0001; interaction F(5, 36) = 2.28, p = .078]*, 7 days later *[c: n = 4 mice per treatment, two‐way ANOVA treatment F(1, 36) = 19.30, p = .0002; layer F(5, 36) = 12.32, p < .0001; interaction F(5, 36) = 0.51, p = .76]* or 14 days later *[d: n = 4 mice per treatment, two‐way ANOVA treatment F(1, 48) = 5.55, p = .027; layer F(5, 48) = 2.38, p = .068; interaction F(5, 48) = 0.17, p = .97]*. Data are presented as mean + *SD*. Asterisks denote significant differences identified by Bonferroni post hoc analysis, *p < .05. Scale bars represent 40 μm. Arrowheads identify YFP^+^ OLIG2^+^ PDGFRα‐negative new oligodendrocytes. White dash lines identify the boundaries of each cortical layer (I–VI) and the underlying corpus callosum (CC) [Color figure can be viewed at http://wileyonlinelibrary.com]

**Figure 3:**
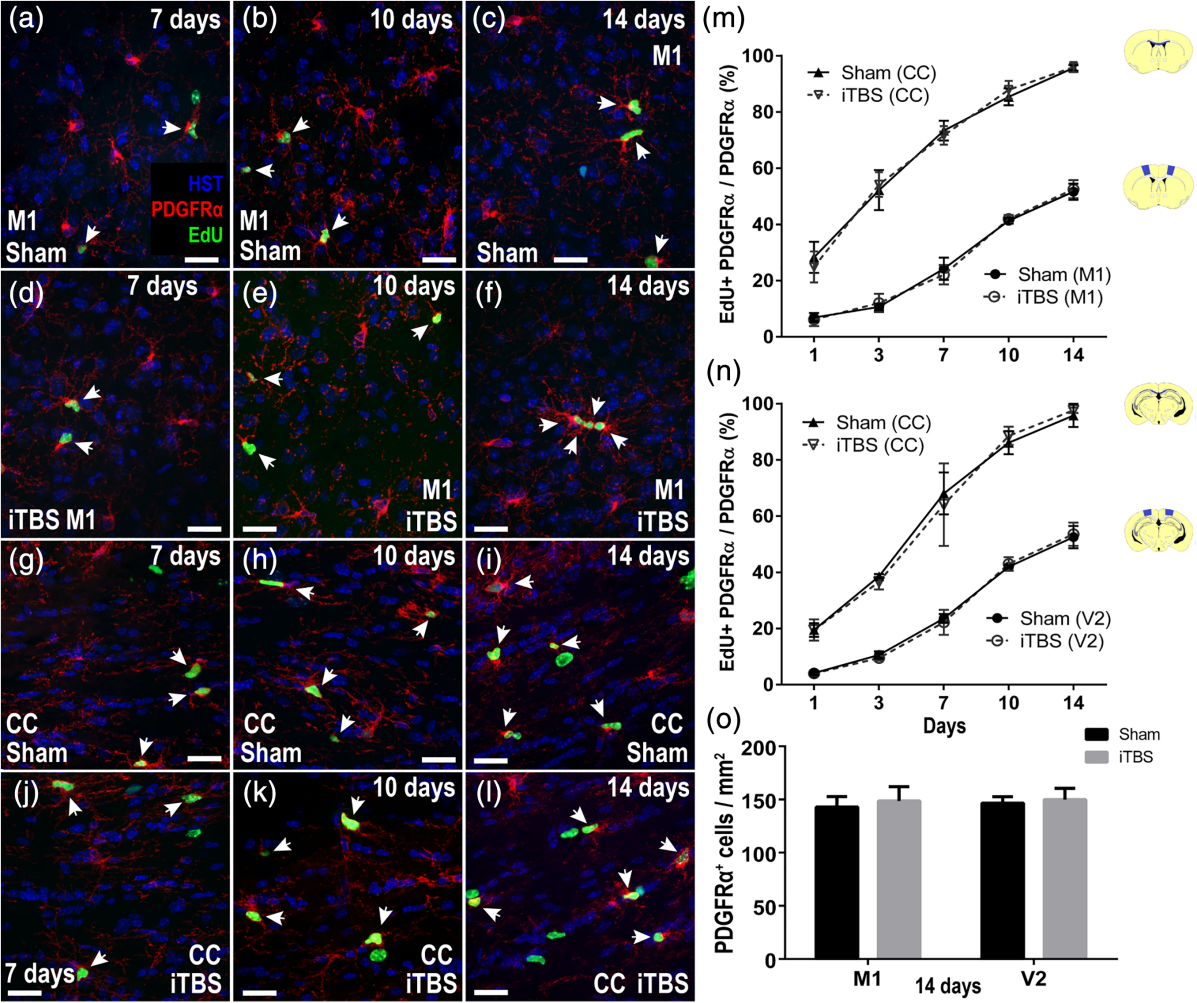
iTBS does not affect OPC proliferation. (a–f) Representative confocal images of EdU (green), PDGFRα (red) and Hoechst 33342 (HST, blue) labeling in the primary motor cortex (M1) and corpus callosum (CC) of mice that received sham (a–c) or iTBS (d–f) treatment alongside EdU administration for 7, 10, or 14 days. (g–l) Representative confocal images of EdU (green), PDGFRα (red), and Hoechst 33342 (HST, blue) labeling in the secondary visual cortex (V2) and corpus callosum (CC) of mice that received sham (g–i) or iTBS (j–l) treatment alongside EdU administration for 7, 10, or 14 days. (m,n) Quantification of the proportion of OPCs that incorporate EdU (EdU^+^ PDGFRα^+^/PDGFRα^+^ × 100) in the M1 cortex *[m: two‐way ANOVA treatment F(1, 20) = 0.001, p = .97; labeling period F(4, 20) = 329.7, p < .0001; interaction F(4, 20) = 0.44, p = .77]* and its underlying CC *[m: two‐way ANOVA treatment F(1, 20) = 0.002, p = .96; labeling period F(5, 20) = 260.4, p < .0001; interaction F(5, 20) = 0.52, p = .71]* or V2 *[n: two‐way ANOVA treatment F(1, 20) = 0.01, p = .90; labeling period F(5, 20) = 402.8, p < .0001; interaction F(5, 20) = 0.29, p = .87]* and its underlying CC *[n: two‐way ANOVA treatment F(1, 20) = 0.02, p = .86; labeling period F(5, 20) = 186.0, p < .0001; interaction: F(5, 20) = 0.31, p = .86]* in sham (solid line) and iTBS (dashed line) mice. (O) Quantification of the density of OPCs (PDGFRα^+^/mm^2^) within the M1 and V2 cortices of mice 1 day after receiving 14 days of sham stimulation (black) or iTBS (grey) *[two‐way ANOVA treatment F(1, 8) = 0.63, p = .44; region F(1, 8) = 0.19, p = .66; interaction F(1, 8) = 0.04, p = .83]*. Data are presented as mean ± *SD* for *n* = 3 mice per treatment group. Scale bars represent 15 μm. Arrows indicate EdU^+^ PDGFRα^+^ recently divided OPCs [Color figure can be viewed at http://wileyonlinelibrary.com]

Data were analyzed per animal and expressed as mean ± standard deviation (*SD*) or per cell and expressed as mean ± standard error of the mean (*SEM*). The number of animals analyzed in each group (*n*) is indicated in the corresponding figure legend. When cell count data were expressed as cell density, the total number of cells counted within the region of interest was divided by the size of the area (defined in x–y coordinates only, as the z‐depth was consistently 30 μm) and expressed as cells per mm^2^ (Figure 1). For data in Figure 2, the total number of newly differentiated oligodendrocytes (YFP^+^ OLIG2^+^ PDGFRα‐neg) was instead expressed as a proportion (%) of all YFP^+^ OLIG2^+^ cells in the region (as per Rivers et al., 2008 and Young et al., 2013). As the vast majority of YFP‐labeled cells are OPCs, this approach allowed the rate of oligodendrogenesis to be standardized to the fraction of YFP‐labeled OPCs within a region to negate any effect of recombination efficiency. Similarly, the proportion of GFP^+^ cells that remained OPCs or had matured into premyelinating or myelinating oligodendrocytes was determined by dividing the number of GFP^+^ OLIG2^+^ PDGFRα‐neg premyelinating or myelinating oligodendrocytes by the total number of GFP^+^ OLIG2^+^ cells in the region (as per Young et al., 2013). When quantifying OPC proliferation (Figure 3), each PDGFRα^+^ OPC was examined for the presence or absence of EdU in the nucleus of the cell, and the data presented as the proportion of OPCs that had incorporated EdU (EdU^+^ PDGFRα^+^/PDGFRα^+^ × 100) (as per Clarke et al., 2012; Young et al., 2013).

All statistical analyses were performed using GraphPad Prism 6 (GraphPad Software). Data were first assessed using the Shapiro–Wilk (*n* > 5) or Kolmogorov–Smirnov (*n* ≤ 5) normality tests and were further analyzed by parametric or nonparametric tests as appropriate. The number of newly formed oligodendrocytes was quantified in mice receiving each stimulation pattern and the effect of stimulation determined using a one‐way ANOVA with a Bonferroni posttest (Figure 1). Normally distributed data with two independent factors (treatment vs. CNS region or treatment duration) were analyzed by two‐way ANOVA with a Bonferroni posttest. ANOVA main effects are given in each figure legend or, where data are not graphically represented, in text. By contrast, internode distribution data were analyzed using a nonparametric Kolmogorov–Smirnov (KS) test for comparison of two sample distributions. Statistical significance was defined as *p* < .05.

## 3 RESULTS

### 3.1 LI‐rTMS increases new oligodendrocyte number in the mouse cortex in a region and pattern specific manner

rTMS frequencies are often described as simple protocols, for example, 10 Hz (10 pulses/s), or patterned protocols, such as theta burst stimulation (three pulses of 50 Hz repeated at 5 Hz), and can be further categorized by their intended effect on neuronal excitability (i.e., excitatory or inhibitory). Typically, simple frequencies >5 Hz and patterned iTBS are considered excitatory and lead to enhanced neuronal activity, while frequencies <5 Hz and continuous theta burst stimulation (cTBS) are considered inhibitory and lead to a dampening of activity within the brain (Hoogendam, Ramakers, & Di Lazzaro, 2010; Tang et al., 2017).

To determine whether a specific pattern of LI‐rTMS could promote new oligodendrocyte addition in the healthy brain, we traced the fate of OPCs in *Pdgfrα‐CreER* ^*T2*^:*: Rosa26‐YFP* transgenic mice (as per Rivers et al., 2008). Tamoxifen was administered at P83 and from P90 mice received LI‐rTMS daily for 14 consecutive days, delivered as a: (a) sham stimulation (no magnetic field generated); (b) patterned iTBS (excitatory); (c) simple 10 Hz stimulation (excitatory); or (d) patterned cTBS (inhibitory) (600 pulses; Figure S1). Coronal brain cryosections spanning Bregma +0.5 to −2.5, corresponding to the brain regions underneath the coil, were immunolabeled to detect yellow fluorescent protein (YFP), platelet‐derived growth factor receptor α (PDGFRα; OPCs), and oligodendrocyte transcription factor 2 (OLIG2; all cells of the oligodendrocyte lineage) (Figure 1). We found that an equivalent fraction of OPCs had recombined to become YFP‐labeled (YFP^+^ PDGFRα^+^ OLIG2^+^ / PDGFRα^+^ OLIG2^+^) in the cortex of sham‐, iTBS‐, 10 Hz‐ and cTBS‐treated mice (Figure S3). These YFP‐labeled OPCs produced new oligodendrocytes (YFP^+^ OLIG2^+^ PDGFRα‐neg) within the primary motor (M1; Figure 1a,b), primary somatosensory (S1; Figure 1c) and secondary visual (V2; Figure 1d) cortices of sham‐stimulated mice. However, a larger number of new YFP^+^ oligodendrocytes were added to the M1 (Figure 1e,f,q,r) and V2 (Figure 1h,t) cortices of iTBS‐treated mice. By contrast, new oligodendrocyte number was not affected by LI‐rTMS when it was delivered as a 10 Hz‐stimulation (Figure 1i–l,q–t) or in a cTBS pattern (Figure 1m–t). For example, a similar number of newly formed oligodendrocytes (YFP^+^ OLIG2^+^ PDGFRα‐neg) accumulated within the V2 cortex of sham (29.11 ± 5.8 cells/mm^2^), 10 Hz (30.99 ± 8.6 cells/mm^2^) and cTBS (26.60 ± 6.2 cells/mm^2^) mice, but significantly more new oligodendrocytes were present in the V2 of iTBS‐treated mice (54.43 ± 12.8 cells/mm^2^, mean ± *SD*; Figure 1t).

These data indicate that LI‐rTMS, delivered in an iTBS pattern, can increase the number of newborn or newly differentiated oligodendrocytes in the adult mouse cortex, but not all cortical regions were equally affected (Table S1; Figure S4). We found that cortical regions underneath the circumference of the coil, such as M1 and V2, contained more new oligodendrocytes in iTBS‐treated mice compared to sham‐treated controls, but that cortical regions underneath the center of the coil, such as S1 (Figure 1c,g,s), were largely unaffected (Table S1; Figure S4). This pattern is consistent with the physical properties of electromagnetic induction, as a circular TMS coil induces a magnetic field that is strongest at its center (Grehl et al., 2015), but the electrical currents induced in brain tissue are maximal under the outer edge of the coil (Hallett, 2007). The implication is that the increase in new oligodendrocyte number is associated with current induction by LI‐rTMS.

To examine the impact that TMS coil position has on new oligodendrocyte number, we again delivered sham and iTBS‐treatment to *Pdgfrα‐CreER* ^*T2*^:*: Rosa26‐YFP* mice, but this time positioned the circular coil over the spinal cord, which is more structurally homogeneous along the rostro‐caudal axis than the brain. We found that iTBS significantly increased the number of new (YFP^+^ OLIG2^+^ PDGFRα‐neg) oligodendrocytes quantified in spinal cord underneath the T13 vertebrae *(Sham: 20.10 ± 3.9 cells/mm* ^*2*^ *, iTBS: 36.11 ± 8.1 cells/mm* ^*2*^ *, mean ± SD)* and the front edge of the coil (Figure S2). However, in spinal cord underneath the L1 vertebrae and the center of the coil, the number of new oligodendrocytes was equivalent between sham‐ and iTBS‐treated mice (Figure S2; *sham: 17.18 ± 3.2 cells/mm* ^*2*^ *, iTBS: 17.72 ± 2.5 cells/mm* ^*2*^ *, mean ± SD*). Our results in brain and spinal cord suggest that LI‐rTMS, delivered as an iTBS pattern, is capable of increasing the number of new oligodendrocytes in the CNS, and that this effect is spatially associated with the location of current rather than magnetic field induction.

### 3.2 LI‐rTMS can increase new oligodendrocyte number in the superficial and deep layers of the cortex

As the intensity of the magnetic field and induced current diminishes with increasing distance from the TMS coil (Tang, Lowe, et al., 2016), more ventral brain regions may be less responsive to stimulation. To determine whether iTBS can increase the number of new oligodendrocytes detected in each cortical layer, we performed a laminar analysis, quantifying the proportion of YFP^+^ OLIG2^+^ cells that were new oligodendrocytes (YFP^+^ OLIG2^+^ PDGFRα‐neg / YFP^+^ OLIG2^+^ x 100; Figure 2). In sham‐stimulated mice, a similar proportion of YFP^+^ OLIG2^+^ cells were newly generated oligodendrocytes in each layer of V2 [Figure 2a,b; *one‐way ANOVA cortical layers I–VI, F(4, 20) = 2.01, p = .16*], suggesting that under normal conditions, oligodendrocyte addition occurs evenly throughout the cortical layers. iTBS treatment increased the proportion of YFP^+^ cells that were OLIG2^+^ PDGFRα‐negative oligodendrocytes in V2 layers I, V and VI (Figure 2a,b), but had no effect on layers II/III, IV or the CC (Figure 2b and Figure S5), suggesting that these layers either accommodate fewer new oligodendrocytes or are refractory to stimulation (Koudelka et al., 2016). As the ability of iTBS to increase new oligodendrocyte number does not decrease linearly from the superficial to deeper layers of the cortex the efficacy of iTBS may be influenced by the cellular and structural features of each brain region in addition to the intensity of the magnetic field and induced current.

### 3.3 LI‐rTMS does not alter OPC proliferation or density

In order to increase the number of new oligodendrocytes in the adult mouse cortex, iTBS could: (a) increase OPC proliferation to increase oligodendrocyte production; (b) increase the number of OPCs that directly differentiate into oligodendrocytes, which would either deplete the OPC pool or trigger the homeostatic replacement of OPCs through increased proliferation (Hughes, Kang, Masahiro, & Bergles, 2013); or (c) enhance the survival of newborn oligodendrocytes. To examine the first possibility, we cumulatively labeled OPCs as they underwent cell division by administering the thymidine analogue, EdU, to mice via their drinking water (Figure 3). EdU‐labeled PDGFRα^+^ OPCs were detected in the M1 cortex (Figure 3a–f,m), CC (Figure 3g–l,m,n) and V2 cortex (Figure 3n) of sham and iTBS‐treated mice at all time‐points examined. However, the fraction of OPCs that became EdU‐labeled was unchanged by iTBS treatment (Figure 3m,n), indicating that iTBS does not increase the number of new oligodendrocytes in the M1 or V2 cortex by increasing OPC proliferation. iTBS additionally had no effect on OPC density (Figure 3o), indicating that it does not increase OPC differentiation.

In the healthy, adult rodent CNS, a small number of apoptotic cells can be detected outside of the neurogenic zones (Dawson, Polito, Levine, & Reynolds, 2003; Ferrer, Bernet, Soriano, Del Rio, & Fonseca, 1990; Hill, Patel, Goncalves, Grutzendler, & Nishiyama, 2014; Hughes et al., 2013; Payne et al., 2013), and in the optic nerve these TUNEL^+^ cells have been shown to co‐label with the oligodendrocyte marker CC1 (Payne et al., 2013). While mature, myelinating oligodendrocytes are long‐lived cells (Hill, Li, & Grutzendler, 2018; Tripathi et al., 2017), only a subset of newborn oligodendrocytes survive to reach maturity. The rate of OPC proliferation significantly outstrips the rate of new oligodendrocyte accumulation in the M1, CC, and optic nerve (Rivers et al., 2008; Young et al., 2013), and live‐imaging of the adult mouse somatosensory cortex has shown that ∼78% of all newborn premyelinating oligodendrocytes die soon after they are generated (Hughes, Orthmann‐Murphy, Langseth, & Bergles, 2018). These data suggest that any intervention that enhances new oligodendrocyte survival could readily increase new oligodendrocyte number over time.

To determine whether 14 days of iTBS could increase new oligodendrocyte number by reducing the level of cell death, we performed TUNEL labeling of coronal cryosections through M1. We found that the number of TUNEL^+^ apoptotic cells in the M1 cortex was significantly reduced in iTBS‐treated mice relative to sham‐treated controls *(sham: 3.36 ± 0.43 cells/mm* ^*2*^ *; iTBS: 1.75 ± 0.60 cells/mm* ^*2*^ *, mean ± SD, t test p = .019, n = 3 mice per treatment group)*. Furthermore, in the underlying CC, where new oligodendrocyte number is not affected by iTBS treatment (Figure 2b; Figure S5), the number of TUNEL^+^ apoptotic cells was equivalent in sham and iTBS‐treated mice *(sham: 4.17 ± 2.30 cells/mm* ^*2*^ *; iTBS: 4.29 ± 2.80 cells/mm* ^*2*^ *, mean ± SD, t test p = .91, n = 3 mice per treatment group)*. These data suggest that iTBS increases new oligodendrocyte number by preventing these cells from undergoing apoptosis.

### 3.4 LI‐rTMS increases the number of new premyelinating and myelinating oligodendrocytes in the cortex

We predicted that if 14 days of iTBS enhanced the survival of YFP^+^ premyelinating oligodendrocytes without affecting the number of YFP^+^ myelinating oligodendrocytes, the effect of LI‐rTMS would be transient, and the number of new cortical oligodendrocytes would return to sham levels after iTBS treatment was concluded. In V2, 14 days of iTBS treatment resulted in more new oligodendrocytes in layers I, V, and VI, relative to sham‐treated mice, however, 7 days post‐stimulation new oligodendrocyte number had largely returned to sham levels, with only layer VI retaining the additional new oligodendrocytes (compare Figure 2b with Figure 2c). By 14 days post‐stimulation the number of new oligodendrocytes in each layer of V2 was equivalent between sham and iTBS treated mice (Figure 2d), suggesting that 2 weeks of iTBS resulted in the accumulation of additional new premyelinating but not stable myelinating oligodendrocytes.

**Figure 4:**
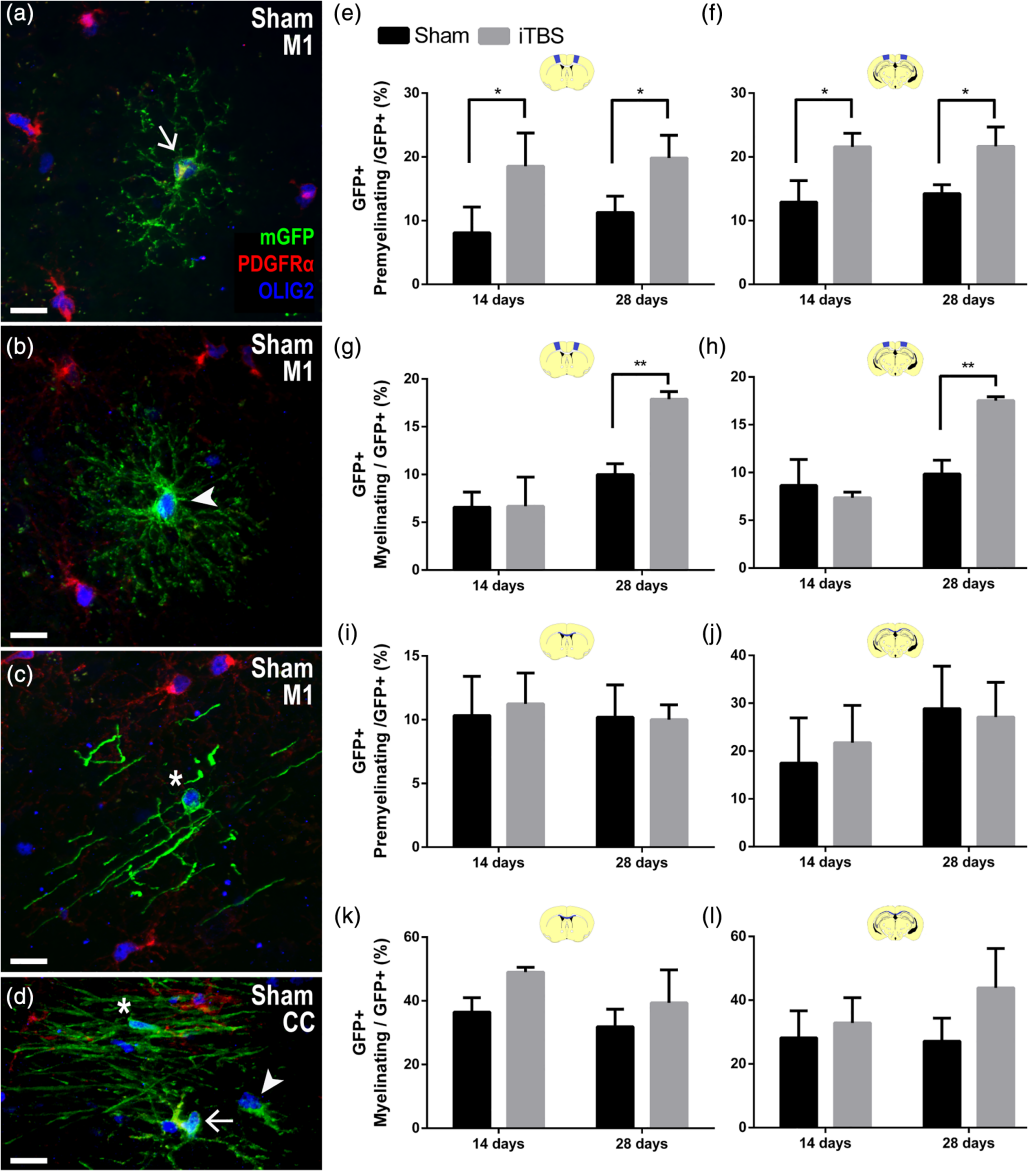
iTBS increases the number of new premyelinating and myelinating oligodendrocytes. (a–c) Representative confocal images from the M1 cortex of sham‐stimulated *Pdgfrα‐CreER* ^*T2*^ *::Tau‐mGFP* mice: (a) a GFP^+^ (green), PDGFRα^+^ (red), OLIG2^+^ (blue) OPC (arrow); (b) a GFP^+^, PDGFRα‐neg, OLIG2^+^ new premyelinating oligodendrocyte (arrowhead), and (c) a GFP^+^, PDGFRα‐neg, OLIG2^+^ new myelinating oligodendrocyte (*). (d) Representative confocal image from the CC of a sham‐stimulated *Pdgfrα‐CreER* ^*T2*^ *::Tau‐mGFP* mouse showing a GFP^+^ OPC (arrow); GFP^+^ new premyelinating oligodendrocyte (arrowhead), and GFP^+^ new myelinating oligodendrocyte (asterisk). (e,f) Graph showing the proportion of GFP^+^ OLIG2^+^ cells that are new premyelinating oligodendrocytes within M1 *[e: two‐way ANOVA treatment F (1, 8) = 18.77, p = .0025; treatment duration F(1, 8) = 1.51, p = .25; interaction F (1, 8) = 0.12, p = .73]* and V2 *[f: two‐way ANOVA treatment F(1, 8) = 34.74, p = .0004; treatment duration F(1, 8) = 0.52, p = .49; interaction F(1, 8) = 0.28, p = .61],* 1 day after 14 or 28 days of sham (black) or iTBS (grey) treatment. (g,h) Quantification of the proportion of GFP^+^ OLIG2^+^ cells that are new myelinating oligodendrocytes within M1 *[g: two‐way ANOVA treatment F(1, 8) = 12.01, p = .01; treatment duration F(1, 8) = 40.10, p = .0004; interaction F(1, 8) = 11.38, p = .01]* and V2 [H, two‐way ANOVA: treatment *F*(1, 8) = 9.99, *p* = .013; treatment duration *F*(1, 8) = 41.07, *p* = .0002; interaction *F*(1, 8) = 21.12, *p* = .0018], 1 day after 14 or 28 days of sham (black) or iTBS (grey) treatment. (i,j) Graph showing the proportion of GFP^+^ OLIG2^+^ cells that are new premyelinating oligodendrocytes within the CC underlying M1 *[i: two‐way ANOVA treatment F(1, 8) = 0.06, p = .80; treatment duration F(1, 8) = 0.24, p = .63; interaction F(1, 8) = 0.15, p = .70]* and the CC underlying V2 *[j: two‐way ANOVA: treatment F(1, 8) = 0.04, p = .84; treatment duration F(1, 8) = 2.06, p = .21; interaction F(1, 8) = 0.26, p = .62],* 1 day after 14 or 28 days of sham (black) or iTBS (grey) treatment. (k,l) Quantification of the proportion of GFP^+^ OLIG2^+^ cells that are new myelinating oligodendrocytes within the CC underlying M1 *[k: two‐way ANOVA treatment F(1, 8) = 3.36, p = .09; treatment duration F(1, 8) = 4.48, p = .06; interaction F(1, 8) = 0.26, p = .62]* and the CC underlying V2 *[l: two‐way ANOVA treatment F(1, 8) = 3.04, p = .14; treatment duration F(1, 8) = 0.65, p = .45; interaction F(1, 8) = 0.96, p = .37]*, 1 day after 14 or 28 days of sham (black) or iTBS (grey) treatment. Data are presented as mean + *SD* for *n* = 3 mice per treatment group. Asterisks denote significant differences identified by Bonferroni post hoc analyses: *p < .05 and **p < .01. Scale bars represent 15 μm [Color figure can be viewed at http://wileyonlinelibrary.com]

To examine this possibility more directly, we followed the maturation of newborn oligodendrocytes in response to sham and iTBS treatment. Tamoxifen was administered to P83 *Pdgfrα‐CreER* ^*T2*^:*: Tau‐mGFP* transgenic mice (as per Young et al., 2013), such that OPCs and the oligodendrocytes they produce express a membrane‐targeted form of green fluorescent protein (GFP), which allowed premyelinating and myelinating oligodendrocytes to be morphologically distinguished in situ (Figure 4). From P90, mice received 14 or 28 consecutive days of sham or iTBS treatment before coronal brain cryosections were immunolabeled to detect GFP, the OPC marker PDGFRα^+^ and the pan‐oligodendrocyte marker OLIG2^+^ (Figure 4a–d). In M1 and V2 a subset of brain OPCs (PDGFRα^+^ OLIG2^+^) became GFP labeled, and the recombination efficiency was equivalent between sham and iTBS treated mice (Figure S3). Consistent with our previous data (see Figure 1), the proportion of GFP^+^ cells that were new oligodendrocytes (PDGFRα‐neg OLIG2^+^) was elevated in M1, but not the underlying CC, of iTBS treated mice relative to sham‐treated controls *[M1 sham: 14.6 ± 2.9%; M1 iTBS: 25.2 ± 5.1%; CC sham: 46.7 ± 7%; CC iTBS: 56.9.2 ± 3.3%; two‐way ANOVA treatment F(1, 8) = 12.70, p = .007; region F(1, 8) = 120.9, p < .0001; interaction F(1, 8) = 0.005, p = .94; Bonferroni posttest M1 sham* vs *iTBS, p = .03; CC sham* vs *iTBS, p = .09*]. The proportion of GFP^+^ cells that were new oligodendrocytes (PDGFRα‐neg OLIG2^+^) was similarly elevated in V2, but not the underlying CC, of iTBS treated mice relative to sham‐treated controls *[V1 sham: 20.8 ± 3.2%; V2 iTBS: 30.5 ± 1.7%; CC sham: 43.2 ± 7.1%; CC iTBS: 53.7 ± 2.4%; two‐way ANOVA treatment F(1, 8) = 9.92, p = .01; region F(1, 8) = 89.46, p < .0001; interaction F(1, 8) = 0.36, p = .56; Bonferroni posttest V2 sham* vs *iTBS, p = .008; V2 CC sham* vs *iTBS, p = .09]*.

A combined histological and morphological analysis of the fate of GFP^+^ OLIG2^+^ cells in the M1 (Figure 4e) and V2 (Figure 4f) cortices, revealed that 14 days of iTBS treatment increased the proportion that were premyelinating oligodendrocytes. However, 14 days of iTBS was insufficient to alter the proportion of GFP^+^ OLIG2^+^ cells that were myelinating oligodendrocytes in either cortical region (Figure 4g,h). When iTBS was instead delivered for 28 consecutive days, the proportion of GFP^+^ OLIG2^+^ cells that had matured into myelinating oligodendrocytes in the M1 (Figure 4g) and V2 (Figure 4h) cortices was also elevated, relative to sham‐treated controls. Oligodendrocyte differentiation and maturation can be a protracted process in the healthy adult mouse cortex (>7 days; Hill et al., 2014; Hughes et al., 2013) and these data suggest that iTBS must be sustained for more than 14 days to increase the number of new myelinating oligodendrocytes added to the cortex.

### 3.5 The length of GFP^+^ newly elaborated internodes is increased by iTBS

**Figure 5:**
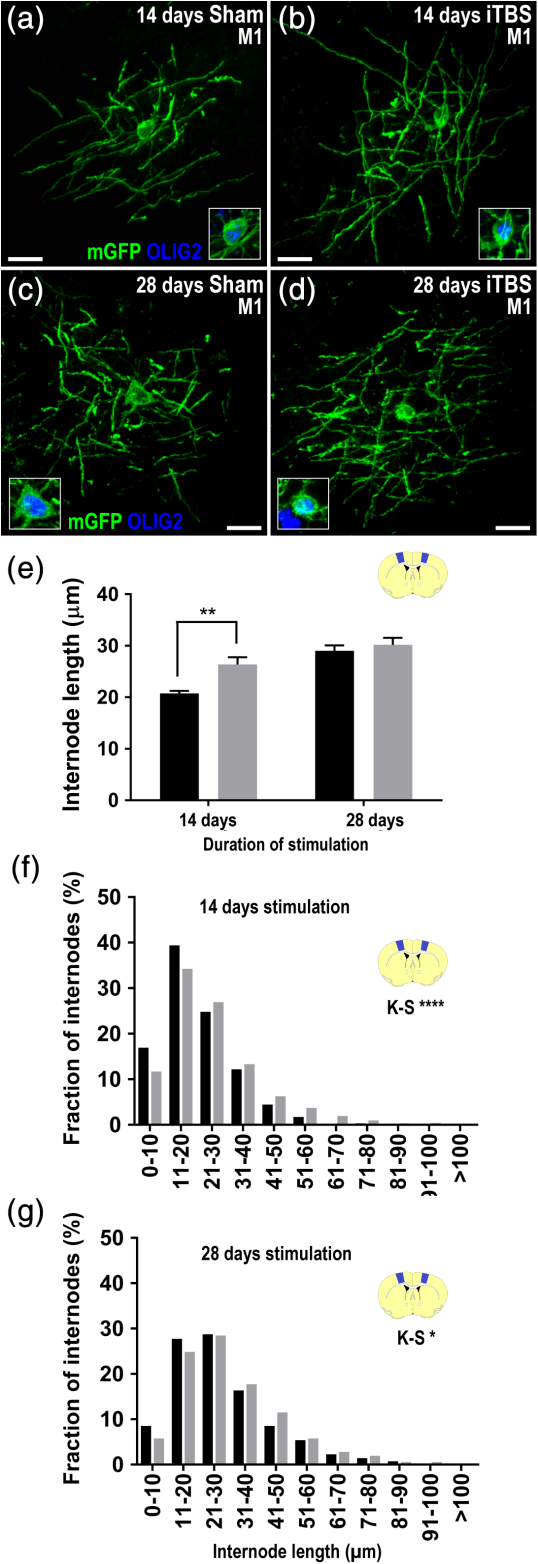
iTBS treatment increases myelin internode length in the M1 cortex. (a–d) High magnification compressed confocal images of new myelinating oligodendrocytes that express GFP (green) and OLIG2 (blue) in M1 after 14 days (a,b) or 28 days (c,d) of sham and iTBS treatment. A single scan through the nucleus of each oligodendrocyte is inset. Scale bars represent 15 μm. (e) Quantification of the average length of GFP^+^ internodes elaborated by individual new myelinating oligodendrocytes in M1 of sham (black bars) or iTBS (grey bars) treated mice after 14 and 28 days of stimulation *[n = 26 sham and n = 39 iTBS GFP* ^*+*^ *oligodendrocytes were analyzed at 14 days and n = 22 sham and n = 23 iTBS GFP* ^*+*^ *oligodendrocytes were analyzed at 28 days; two‐way ANOVA treatment F(1, 106) = 7.14, p = .008; treatment duration F(1, 106) = 22.53, p < .0001; interaction F(1, 106) = 3.092, p = .08]*. Data presented as mean + *SEM* and asterisks denote a significant difference identified by Bonferroni post hoc analysis, **p < .01. (f–g) Graphical representation of internode length distribution for GFP^+^ internodes elaborated in the M1 cortex over 14 days *(f: n = 997 sham and n = 1,175 iTBS GFP* ^*+*^ *new internodes; K–S test D = 0.127, p < .0001)* or 28 days *(g: n = 703 sham and n = 885 iTBS GFP* ^*+*^ *new internodes; K–S test D = 0.08, p = .017)* of sham or iTBS treatment. Internodes were measured across *n* = 3 mice per treatment group [Color figure can be viewed at http://wileyonlinelibrary.com]

**Figure 6:**
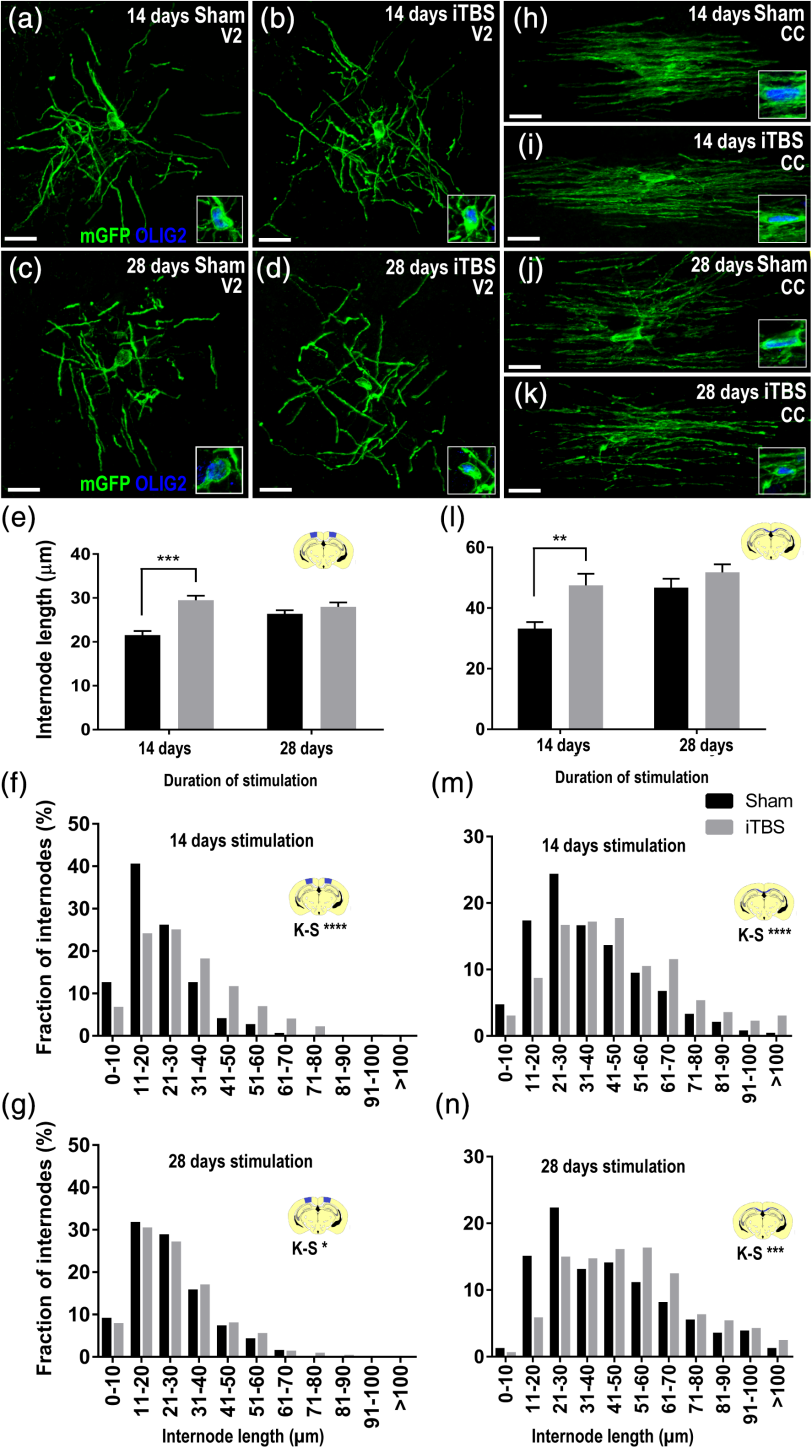
iTBS increases new myelin internode length in the V2 cortex and underlying CC. (a–d) High magnification compressed confocal images of new myelinating oligodendrocytes expressing GFP (green) and OLIG2 (blue) in V2 after 14 days (a,b) and 28 days (c,d) of sham and iTBS treatment. A single scan through the nucleus of each oligodendrocyte is inset. (e) Quantification of the average length of GFP^+^ internodes elaborated by individual new myelinating oligodendrocytes in V2 of sham (black bars) or iTBS (grey bars) mice after 14 and 28 days of stimulation *[n = 18 sham and n = 19 iTBS GFP* ^*+*^ *oligodendrocytes were analyzed at 14 days and n = 16 sham and n = 16 iTBS GFP* ^*+*^ *oligodendrocytes were analyzed at 28 days; two‐way ANOVA treatment F(1, 65) = 24.04, p < .0001; treatment duration F(1, 65) = 2.95, p = .08; interaction F(1, 65) = 10.74, p = .0017]*. Data are presented as mean + *SEM* and oligodendrocytes were sampled from *n* = 3 mice per treatment group. Asterisks denote significant differences identified by Bonferroni post hoc analysis, **p < .01, ***p < .001. (f,g) Graphical representation of internode length distribution for GFP^+^ new internodes elaborated in the V2 cortex within 14 days *(F: n = 575 sham and n = 676 iTBS GFP* ^*+*^ *internodes; K–S test D = 0.23, p < .0001)* and 28 days *(G: n = 546 sham and n = 605 iTBS GFP* ^*+*^ *internodes; K–S test D = 0.08, p = .04)* of sham (black bars) or iTBS (grey bars) treatment. (h–k) High magnification compressed confocal images of new myelinating oligodendrocyte, expressing GFP (green) and OLIG2 (blue) in the corpus callosum (CC) underlying V2 following 14 days (h,i) or 28 days (j,k) of sham or iTBS treatment. (l) Quantification of the average length of internodes elaborated by individual new myelinating oligodendrocytes in the CC underlying V2 after 14 and 28 days of sham or iTBS treatment *[n = 17 sham and n = 14 iTBS new oligodendrocytes analyzed at 14 days and n = 11 sham and n = 12 iTBS new oligodendrocytes analyzed at 28 days; two‐way ANOVA treatment F(1, 50) = 10.48, p = .0021; treatment duration F(1, 50) = 8.95, p = .0043; interaction F(1, 50) = 2.37, p = .12]*. (m,n) Graphical representation of internode length distribution for GFP^+^ new internodes elaborated in the CC underlying V2 within 14 days *(m: n = 840 sham and n = 400 iTBS GFP* ^*+*^ *internodes; K–S test D = 0.19, p < .0001)* or 28 days *(n: n = 306 sham and n = 440 iTBS GFP* ^*+*^ *internodes; K–S test D = 0.18, p < .001)* of sham (black bars) or iTBS (grey bars) treatment. Scale bars represent 15 μm. Oligodendrocytes and internodes were sampled across *n* = 3 mice per treatment group [Color figure can be viewed at http://wileyonlinelibrary.com]

To determine whether iTBS could influence the number or length of internodes elaborated by new myelinating cells, we first analyzed the morphology of individual GFP^+^ OLIG2^+^ myelinating oligodendrocytes in the M1 (Figure 5) and V2 (Figure 6) cortices of mice that had received 14 or 28 days of sham or iTBS treatment. We found that iTBS did not alter the number of internodes elaborated by new myelinating M1 oligodendrocytes (Figure S6). However, after 14 days of iTBS, new myelinating oligodendrocytes produced internodes that were, on average, longer in iTBS‐treated mice than sham‐treated controls (Figure 5a,b,e). Our analysis of GFP^+^ M1 internode length distribution also revealed that internodes added over 14 or 28 days of treatment were longer in iTBS than sham treated mice (Figure 5a–d,f–g). Fourteen days of iTBS similarly affected the morphology of GFP^+^ new myelinating oligodendrocyte in the V2 cortex, which elaborated internodes that were longer, on average, than those elaborated under sham conditions (Figure 6a,b,e). Furthermore, our analysis of GFP^+^ V2 internode length distribution indicated that both 14 and 28 days of iTBS was associated with the elaboration of longer internodes (Figure 6a–d,f,g).

The ability of iTBS to influence internode length appears to be independent of its capacity to increase new oligodendrocyte survival and number. We report that 14 or 28 days of iTBS had no effect on the number of new premyelinating or myelinating oligodendrocytes in the CC (see Figure 4i–l), yet 14 days of iTBS still increased the average length of the GFP^+^ internodes produced by new myelinating oligodendrocytes added to this region (Figure 6h,i,l)). Furthermore, 14 and 28 days of iTBS produced a significant right‐ward shift in GFP^+^ internode length distribution (Figure 6j,k,m,n), indicative of longer internodes. As new oligodendrocyte survival and number are not affected by iTBS in the CC, but internode length is still increased, it follows that iTBS can promote myelinating oligodendrocyte maturation by a different mechanism—perhaps by increasing the rate of internode elaboration or by increasing the final length of internodes produced.

## 4 DISCUSSION

rTMS is being evaluated in a growing number of clinical contexts, and while the cellular changes that underpin the behavioral and therapeutic outcomes are largely unknown, they are likely to comprise neuronal and glial responses (Cullen & Young, 2016). This study shows that LI‐rTMS, applied in an iTBS pattern, can increase the number of new oligodendrocytes that are incorporated into the cortex over time (Figure 1 and Figure 4). The effect of iTBS on new oligodendrocyte number was only seen underneath the circumference of the coil (Table S1 and Figure S4), in brain regions where current induction is strongest (Deng, Lisanby, & Peterchev, 2013). Furthermore, iTBS did not increase new oligodendrocyte number by altering OPC proliferation or differentiation, which would have influenced EdU incorporation or OPC density (Figure 3). Instead, iTBS enhanced cell survival in the cortex. Consequently, 2 weeks of iTBS increased the number of premyelinating oligodendrocytes present in the M1 and V2 cortices, and 4 weeks of iTBS saw these cells mature further into new myelinating oligodendrocytes (Figure 4). By analyzing the morphology of differentiating oligodendrocytes, we determined that iTBS increased the length of internodes supported by maturing oligodendrocytes in the M1 and V2 cortices, as well as the CC (Figures 5 and 6). As new oligodendrocyte survival and number were not modified by iTBS in the CC, iTBS must independently effect myelinating oligodendrocyte maturation, perhaps by increasing their rate of internode elaboration or final internode length. The ability of LI‐rTMS to enhance new oligodendrocyte survival and increase the number of mature, myelinating oligodendrocytes added to the healthy brain, suggests that it would be worth exploring the ability of this technique to promote remyelination.

### 4.1 iTBS does not increase oligodendrogenesis

LI‐rTMS, delivered as an iTBS pattern, increased the number of new oligodendrocytes detected in the cortical grey matter, but had no effect when delivered at 10 Hz or as a cTBS pattern (Figure 1). Furthermore, iTBS produced this effect without influencing oligodendrogenesis (OPC proliferation or differentiation; Figure 3). This outcome was unexpected, as both 10 Hz and iTBS are predicted to increase neuronal activity, which is a potent modulator of OPC proliferation and differentiation within the CNS (reviewed by Mount & Monje, 2017). For example, a 5 Hz stimulation of the CC in freely behaving mice (single 3 hr session via implanted electrodes), can promote OPC differentiation without significantly increasing proliferation (Nagy, Hovhannisyan, Barzan, Chen, & Kukley, 2017). By contrast a 25 Hz stimulation of the CC or a 20 Hz optogenetic stimulation of M1 layer V pyramidal neurons (30 min daily for 7 days), triggers OPC proliferation and increases the number of newborn CC1^+^ oligodendrocytes (Gibson et al., 2014; Nagy et al., 2017). A 300 Hz stimulation of the CC (Nagy et al., 2017) or 333 Hz stimulation of corticospinal tract neurons (6 hr a day, over 10 days; (Li et al., 2010) similarly promotes OPC proliferation and oligodendrogenesis.

Unlike these direct stimulation methods, where the frequency of neuronal firing closely mirrors the frequency of stimulation, LI‐rTMS does not directly elicit neuronal firing. When the rodent coil was used to deliver iTBS to ex vivo cortical slices, it increased neuronal excitability—not by directly triggering action potentials, but by lowering the action potential threshold and increasing the evoked spike firing frequency immediately after stimulation and for at least 20 min post‐stimulation (Tang, Hong, et al., 2016). While these data support the ability of iTBS to increase neuronal firing, our data suggest that the net effect of iTBS on network activity in vivo is not comparable to direct stimulation at 5, 20, or 300 Hz, as iTBS did not modulate OPC proliferation or direct differentiation.

### 4.2 iTBS promotes the survival of premyelinating oligodendrocytes

LI‐rTMS, delivered as an iTBS pattern for 14 days, increased the number of newborn, premyelinating oligodendrocytes present in the M1 and V2 cortices (Figure 4), and our cre‐lox lineage tracing approach confirmed that these cells were the progeny of parenchymal OPCs and not neural stem cells (Cavaliere, Benito‐Munoz, Panicker, & Matute, 2013). This increase in new oligodendrocyte number was not associated with an increase in OPC proliferation or a change in OPC density, suggesting that new oligodendrocyte number was increased due to enhanced cell survival.

It is well established that in the developing and adult rodent brain, not all newborn oligodendrocytes survive to reach maturity. In the postnatal mouse optic nerve ∼50% of oligodendrocytes die by P14 (Barres et al., 1992) and in the developing neocortex ∼20% of premyelinating cells die between P7 and P11 (Trapp, Nishiyama, Cheng, & Macklin, 1997). In the adult mouse brain and optic nerve, OPCs continuously proliferate to generate new oligodendrocytes, and the level of OPC proliferation far exceeds the number of new oligodendrocytes that accumulate over time (Rivers et al., 2008; Young et al., 2013). From these data, it has been estimated that only ∼41% of optic nerve and ∼30% of callosal adult‐generated oligodendrocytes survive long term (Young et al., 2013) and recent evidence has shown that in the somatosensory cortex only ∼22% of newly differentiated oligodendrocytes survive to become fully integrated myelinating cells (Hughes et al., 2018). These numbers suggest that oligodendrocytes are the major cell type undergoing apoptosis outside of the neurogenic zones in the healthy adult rodent CNS, and all apoptotic cells detected in the healthy adult rat optic nerve are confirmed CC1^+^ oligodendrocytes (Payne et al., 2013).

Despite the seemingly large number of premyelinating oligodendrocytes that are generated and die, the number of apoptotic cells that can be identified in the adult mouse cortex is very small. We detected only ∼3.36 TUNEL^+^ apoptotic cells per mm^2^ of the M1 cortex in sham‐stimulated mice. This is consistent with previous reports (Dawson et al., 2003; Ferrer et al., 1990), and reflects the rapid clearance of apoptotic cells from the brain (Thomaidou, Mione, Cavanagh, & Parnavelas, 1997), such that very few dying cells can be identified at any single time‐point. However, we observed that iTBS treatment significantly reduced the number of apoptotic cells present in the cortex to ∼1.75 TUNEL^+^ apoptotic cells per mm^2^, a reduction in cell death that could cumulatively, over a 2 week period, account for more new oligodendrocytes in the cortex of iTBS‐treated mice.

iTBS could act directly on premyelinating oligodendrocytes to influence their survival, as the transcription factor EB can turn on expression of *Bbc3* (Bcl binding component 3) and activate Bax‐Bak signaling to trigger apoptotic cell death (Sun et al., 2018). However, it is also possible that this effect is secondary to an increase in neuronal activity (Kougioumtzidou et al., 2017; Palser, Norman, Saffell, & Reynolds, 2009; Trapp et al., 1997; Xiao et al., 2016). More specifically, glutamatergic signaling can support premyelinating oligodendrocyte survival, as the conditional deletion of AMPA receptor subunits from developmental OPCs results in mice having ∼20% fewer premyelinating oligodendrocytes (Enpp6^+^ cells) and ∼20‐27% fewer CC1^+^ oligodendrocytes in the CC by P14 (Kougioumtzidou et al., 2017). This effect was not attributed to a change in OPC proliferation, but rather increased death of newborn oligodendrocytes (Kougioumtzidou et al., 2017). Therefore, iTBS, which can increase glutamatergic signaling (Hoppenrath & Funke, 2013; Labedi, Benali, Mix, Neubacher, & Funke, 2014), could promote premyelinating oligodendrocyte survival through increased AMPA receptor activation.

Additionally, the release of neurotrophins from axons or astrocytes could account for the pro‐survival effect of iTBS. BDNF signaling is important for myelination (Wong et al., 2013; Xiao et al., 2010) and its expression is increased by rTMS (Castillo‐Padilla & Funke, 2016; Müller et al., 2000; Zhang et al., 2015), including LI‐rTMS (Makowiecki et al., 2014). BDNF appears to have no effect on the survival of OPCs (Xiao et al., 2010), but can interact with other trophic factors to enhance the survival of oligodendrocytes (Barres et al., 1993). rTMS may also directly or indirectly stimulate astrocytes to release leukemia inhibitory factor (Cohen & Fields, 2008; Ishibashi et al., 2006), which is another regulator of oligodendrocyte survival and myelination (Gard, Burrell, Pfeiffer, Rudge, & Williams, 1995; Ishibashi et al., 2006).

### 4.3 iTBS increases myelin internode length

Internode length and the number of internodes elaborated by individual oligodendrocytes differs between CNS regions (Butt, Colquhoun, Tutton, & Berry, 1994; Chong et al., 2012; Murtie, Macklin, & Corfas, 2007; Osanai et al., 2017; Tripathi et al., 2017; Young et al., 2013), and is determined by a combination of signals that are both intrinsic and extrinsic to the developing oligodendrocyte (Almeida, Czopka, & Lyons, 2011; Bechler, Byrne, & Ffrench‐Constant, 2015; Hines et al., 2015; reviewed by Bechler et al., 2017). Extrinsic signals, such as increased NogoA‐signaling (Chong et al., 2012), decreased neurotransmitter release (Mensch et al., 2015) and social isolation, which reduces Neuregulin‐ErbB3 receptor signaling (Makinodan, Rosen, Ito, & Corfas, 2012), can reduce the number of myelin internodes elaborated by individual oligodendrocytes. Conversely, the electrical stimulation of dorsal root ganglion neurons in vitro, can double the number of internodes generated by co‐cultured oligodendrocytes (Malone et al., 2013). While iTBS did not change the number of internodes elaborated by individual oligodendrocytes (Figure S5), it did influence oligodendrocyte maturation and myelination, as the resulting myelinating oligodendrocytes supported longer internodes when subjected to iTBS (Figures 5 and 6).

By analyzing newly myelinating oligodendrocytes in the M1 and V2 cortices of 14 day sham and iTBS treated mice, we determined that oligodendrocytes maturing under the influence of iTBS support internodes that are, on average, ∼28 and ∼37% longer than those supported by oligodendrocytes developing under sham conditions. As these cells encompass myelinating oligodendrocytes at various stages of maturation, we also compared the distribution of internode lengths elaborated by the population and found that after 14 or 28 days of stimulation, new internodes were longer in iTBS treated mice than sham stimulated mice. While this effect could be secondary to the effect of iTBS on new oligodendrocyte survival, this seems unlikely as iTBS also increased new internode length in the CC, a region where iTBS failed to influence new oligodendrocyte survival or number. iTBS may instead enhance the maturation of myelinating oligodendrocytes by increasing their rate of internode extension or increasing final internode length. We observed a reduced effect of iTBS on internode length with increasing treatment duration (compare Figure 5f with Figure 5g and compare Figure 6f with Figure 6g), which may suggest that iTBS increases the rate of extension, but does not impact final internode length; however, these possibilities can only be directly investigated by performing live two‐photon imaging of the maturing cells.

There are a number of signals that could be modified by iTBS to increase internode length. For example, GABAergic signaling in neocortical slices, cultured from P8 mice, is associated with increased internode length (Hamilton et al., 2017), however, iTBS also increased internode length in the CC, suggesting that longer internodes may instead result from increased glutamate‐induced calcium signaling in the developing myelin sheaths (Baraban, Koudelka, & Lyons, 2018; Krasnow, Ford, Valdivia, Wilson, & Attwell, 2018). While iTBS could lengthen internodes by modulating neurons, the alternative is that it has a direct effect on the extending internodes of maturing oligodendrocytes, perhaps influencing local calcium signaling (Grehl et al., 2015), and further research is required to dissect the primary and secondary effects of iTBS on these cells.

### 4.4 Future directions

TMS is currently used for the clinical diagnosis of motor neuron disease (Vucic, Ziemann, Eisen, Hallett, & Kiernan, 2013) and MS (Caramia et al., 2004). Furthermore, rTMS is an approved treatment for pharmacologically resistant depression (Rossi, Hallett, Rossini, & Pascual‐Leone, 2009) and has been shown to ameliorate spasticity and fatigue in people with MS (Mori et al., 2010; Mori et al., 2011; Nielsen et al., 1996). As LI‐rTMS can non‐invasively promote new oligodendrocyte survival and maturation in the healthy brain, it may find application in the treatment of hypomyelination or demyelination. In particular, LI‐rTMS may be a suitable adjunct to current pharmacological treatments for MS, as impaired oligodendrocyte survival and maturation are key factors that contribute to remyelination failure in this disease (Franklin & Ffrench‐Constant, 2008).

## CONFLICT OF INTEREST

The authors declare no potential conflict of interest.

## AUTHOR CONTRIBUTIONS

K.M.Y., C.L.C., J.R., and A.D.T. developed the project and wrote the manuscript. C.L.C., M.T.C., M.S., M.E.O., A.D.T., L.A., and K.M.Y. carried out the experiments. K.M.Y., J.R., and C.L.C. obtained the funding. C.L.C. and M.T.C. performed the statistical analyses and generated the figures. K.M.Y., J.R., and C.L.C. provided supervision.

## Supporting information


**Figure S1** Administration of LI‐rTMS to mice using a specialised rodent coil
(A) Photograph of the 8 mm circular rodent coil used to deliver LI‐rTMS to mice in this study (Tang et al., 2016).
(B) Schematic showing the method of restraint (plastic body contour shape restraint bag; pink), placement of the coil (red circle) and approximate region where the greatest current is generated (red circle).
(C) Illustration of the magnetic flux generated by the coil. Image approximately to scale.
(D) Illustration of the 10 Hz, intermittent theta burst (iTBS) and continuous theta burst (cTBS) stimulation patterns delivered in this study. Each bar represents a magnetic pulse that generates a reciprocal pulsed current in the underlying brain. Mice received a total of 600 pulses delivered in one of these patterns, or no pulses, i.e., sham stimulation, where the coil was not activated. This treatment was repeated daily for up to 28 days.

**Figure S2** iTBS increases the number of new oligodendrocytes in the adult mouse spinal cord
(A) P83 *Pdgfrα‐CreER* ^*T2*^ *::Rosa26YFP* transgenic mice received 300 mg/kg Tamoxifen for four consecutive days. At P90, mice were randomly assigned to receive either sham or iTBS stimulation of the spinal cord, daily for 14 consecutive days. For spinal cord stimulation, the coil (red circle) was held over the spinal cord so that the front of the coil was positioned over the T13 vertebrae. Mice were perfusion fixed and the spinal cord collected for immunohistochemical analysis 1 day after treatment cessation.
(B‐E) Low magnification confocal images of transverse spinal cord cryosections (30 μm) collected at the level of the T13 (B‐C; underneath the circumference of the coil) or L1 (D‐E; underneath the center of the coil) vertebrae of sham and iTBS mice. Sections were immunolabeled to detect PDGFRα (red), YFP (green) and OLIG2 (blue).
(F) Graphical representation of YFP^+^, PDGFRα‐negative, OLIG2^+^ new oligodendrocyte number in T13 and L1 spinal cord 1 day after 14 days of sham (black) or iTBS (grey) treatment. iTBS significantly increased new oligodendrocyte number in the T13 spinal cord but had no effect on new oligodendrocyte number in the L1 spinal cord *[two‐way ANOVA treatment F (1,8) = 8.32 p = 0.02; spinal cord region F (1,8) = 13.79, p = 0.0059; interaction F (1,8) = 7.278, p = 0.027]*. Asterisks denote statistical significance identified by Bonferroni post hoc analysis, **p < 0.01.

**Figure S3** An equivalent proportion of the OPC population became fluorescently labeled in sham and LI‐ rTMS‐treated mice
(A‐D) P83 *Pdgfrα‐CreER* ^*T2*^ *::Rosa26YFP* transgenic mice received 300 mg/kg Tamoxifen for four consecutive days. At P90, mice were randomly assigned to a treatment group, receiving either sham stimulation, iTBS, 10 Hz or cTBS, daily for 14 consecutive days. Mice were perfusion fixed for immunohistochemical analysis 1 day after treatment cessation. The proportion of OPCs (PDGFRα^+^) that had undergone recombination to express yellow fluorescent protein (YFP) was quantified in the primary motor cortex (M1) at Bregma +0.5 *[A: treatment F (3,16) = 0.05, p = 0.98]* and Bregma −0.5 *[B: treatment F (3,16) = 0.002, p = 0.99]*, the primary somatosensory cortex (S1) at Bregma −1.5 *[C: treatment F (3,16) = 0.41, p = 0.74]* and the secondary visual cortex (V2) at Bregma −2.5 *[D: treatment F (3,16) = 0.13, p = 0.93]* and is represented graphically. Data are expressed as mean + *SD* for *n* = 4 mice per treatment group. Data were analyzed by a 1‐way ANOVA. No significant difference was detected in the proportion of OPCs that recombined in any treatment group.
(E‐F) P83 *Pdgfrα‐CreER* ^*T2*^ *::Tau‐mGFP* transgenic mice received 300 mg/kg Tamoxifen for four consecutive days. At P90, mice were randomly assigned to a treatment group, receiving either sham stimulation or iTBS for 14 or 28 consecutive days. Mice were perfusion fixed for immunohistochemical analysis 1 day after treatment cessation. The proportion of OPCs (PDGFRα^+^) that express green fluorescent protein (GFP) was quantified in M1 at Bregma +0.5 *[E: treatment F (1,8) = 0.09, p = 0.76; treatment duration F (1,8) = 1.97, p = 0.19; interaction F (1,8) = 0.01, p = 0.89]* and V2 at Bregma −2.5 *[F: treatment F (1,8) = 0.01, p = 0.91, duration: F (1,8) = 0.18, p = 0.68, interaction: F (1,8) = 0.01, p = 0.90]* and is graphically represented. Data are expressed as mean + *SD* for *n* = 3 mice per treatment group. Data were analyzed by a two‐way ANOVA and no significant difference was detected in the level of recombination of any treatment group or duration.

**Figure S4** Visual representation of new oligodendrocyte number in iTBS mice relative to sham stimulated controls at distinct Lateral and Bregma co‐ordinates
(A) P83 *Pdgfrα‐CreER* ^*T2*^ *::Rosa26YFP* transgenic mice received 300 mg/kg Tamoxifen for 4 consecutive days. At P90, mice were randomly assigned to a treatment group, receiving either sham stimulation or iTBS, daily for 14 consecutive days. Mice were perfusion fixed for immunohistochemical analysis 1 day after treatment cessation. Coronal brain sections were collected from anatomically defined regions underneath the electromagnetic coil, and immunohistochemistry performed to quantify the density of YFP^+^ PDGFRα‐negative OLIG2^+^ new oligodendrocytes in each region. As the electromagnetic coil was circular, and there was no obvious difference between the right and left hemispheres, data for each hemisphere was combined to obtain a single density measure for newborn oligodendrocytes that could be mapped to a single anatomical point corresponding to a Bregma level (z‐axis) and lateral distance from the midline (x‐axis). To develop a visual representation of the effect of iTBS, the density of new oligodendrocytes detected in iTBS mice was divided by the density of new oligodendrocytes in sham stimulated mice, to obtain a relative fold change in oligodendrogenesis (y‐axis) at each anatomical point. Blue bars indicate regions of the brain where new oligodendrocyte density was equivalent between sham and iTBS treated mice (fold change of 0.95‐1.49). Red bars indicate brain regions, where the density of new oligodendrocytes in iTBS‐treated mice was ≥1.5 fold higher than sham‐stimulated mice. Fold change was calculated from the average data of *n* = 5 mice per treatment group provided in Table S1.
(B) The graph depicted in (A) has been rotated to more clearly illustrate the location of brain regions in which iTBS had no effect on new oligodendrocyte density (blue) and the location of regions where iTBS increased new oligodendrocyte density relative to sham‐stimulated mice. The location of the red bars form an approximate semi‐circle, that corresponds to the location of the outer circumference of the TMS coil. The cluster of blue bars between Bregma levels −0.5 and − 1.5 closely approximates the position of the center of the TMS coil.
* Please note ‐ the coil is manually held over the head each day, lined up with the front of the ears. The mouse has some capacity to move during the stimulation period, and the operator moves the coil accordingly to ensure it remains in the correct position throughout the stimulation. For this reason, the placement of the TMS coil is not exact and will vary slightly from day to day across the 14 day stimulation period.

**Figure S5** iTBS does not increase new oligodendrocyte number in all M1 cortical layers
(A) Low magnification confocal images of the primary motor cortex (M1) of *Pdgfrα‐CreER* ^*T2*^ *::Rosa26‐YFP* mice that were perfused 1 day after they received 14 days of sham stimulation (left) or iTBS (right), stained to detect PDGFRα (red), YFP (green) and Hoechst 33342 (HST; blue).
(B‐D) Graph showing the proportion of YFP^+^ cells that are newly differentiated oligodendrocytes (PDGFRα‐negative, OLIG2^+^) in each layer of M1 and the CC of mice that received 14 days of sham or iTBS treatment and were perfusion fixed for analysis 1 later *[B: n = 5 mice per treatment, two‐way ANOVA treatment F (1, 48) = 13.79, p = 0.0005; layer F (5, 48) = 6.44, p = 0.0001; interaction F (5, 48) = 1.532, p = 0.19]*, 7 days later *[C: n = 4 mice per treatment, two‐way ANOVA treatment F (1, 36) = 5.40, p = 0.028; layer F (5, 36) = 10.79, p < 0.0001; interaction F (5, 36) = 0.39, p = 0.84]* or 14 days later *[D: n = 4 mice per treatment, two‐way ANOVA treatment F (1, 36) = 11.77, p = 0.0018; layer F (5, 36) = 11.26, p < 0.0001; interaction F (5, 36) = 1.73, p = 0.15]*.
Data are presented as mean + *SD*. Asterisks denote significant differences identified by Bonferroni post hoc analysis, *p < 0.05. Scale bars represent 40 μm. Arrowheads identify YFP^+^ OLIG2^+^ PDGFRα‐negative new oligodendrocytes. White dash lines identify the boundaries of each cortical layer (I‐VI) and the underlying corpus callosum (CC).

**Figure S6** iTBS does not influence the number of internodes elaborated by differentiating oligodendrocytes
(A‐D) P83 *Pdgfrα‐CreER* ^*T2*^ *::Tau‐mGFP* transgenic mice received 300 mg/kg Tamoxifen for 4 consecutive days. At P90, mice were randomly assigned to a treatment group, receiving either sham stimulation or iTBS for 14 or 28 consecutive days. Mice were perfusion fixed for immunohistochemical analysis 1 day after treatment cessation. The number of internodes elaborated by individual GFP^+^ OLIG2^+^ new oligodendrocytes with myelinating morphology was quantified in the primary motor cortex (M1) *[A: treatment F (1, 106) = 3.14, p = 0.08; treatment duration F (1, 106) = 0.73, p = 0.39; interaction F (1, 106) = 0.28, p = 0.59]* and its underlying corpus callosum (C) *[B: treatment F (1, 58) = 1.81, p = 0.18; treatment duration F (1, 58) = 0.36, p = 0.55; interaction F (1, 58) = 1.48, p = 0.31]* or the secondary visual cortex (V2) *[C: treatment F (1, 65) = 0.46, p = 0.49; treatment duration F (1, 65) = 3.61, p = 0.07; interaction F (1, 65) = 3.46, p = 0.08]* and its underlying CC *[D: treatment F (1, 50) = 1.70, p = 0.19; treatment duration F (1, 50) = 3.43, p = 0.07; interaction F (1, 50) = 0.31, p = 0.57]*. Data are expressed as mean + *SEM* and were analyzed by two‐way ANOVA. Oligodendrocytes were imaged from *n* = 3 mice per treatment group.

**Table S1** LI‐rTMS increases regional oligodendrocyte addition.

## References

[glia23620-bib-0001] Almeida, R. G. , Czopka, T. , & Lyons, D. A. (2011). Individual axons regulate the myelinating potential of single oligodendrocytes in vivo. Development, 138(20), 4443–4450. 10.1242/dev.071001 21880787 PMC3177314

[glia23620-bib-0002] Baraban, M. , Koudelka, S. , & Lyons, D. A. (2018). Ca^2+^ activity signatures of myelin sheath formation and growth in vivo. Nature Neuroscience, 21(1), 19–23. 10.1038/s41593-017-0040-x 29230058 PMC5742537

[glia23620-bib-0090] Barker, A. , Freeston, I. , Jalinous, R. , Merton, P. , & Morton, H. (1985). Magnetic Stimulation of the Human Brain. Journal of Physiology (London), 369, P3–P3.

[glia23620-bib-0003] Barres, B. , Hart, I. , Coles, H. , Burne, J. , Richardson, W. D. , & Raff, M. C. (1992). Cell death and control of cell survival in the oligodendrocyte lineage. Cell, 70, 31–46. 10.1016/0092-8674(92)90531-G 1623522

[glia23620-bib-0004] Barres, B. , Jacobson, M. , Schmid, R. , Sendtner, M. , & Raff, M. (1993). Does oligodendrocyte survival depend on axons? Current Biology, 3, 489–497. 10.1016/0960-9822(93)90039-Q 15335686

[glia23620-bib-0005] Barres, B. , & Raff, M. (1993). Proliferation of oligodendrocyte precursor cells depends on electrical activity in axons. Nature, 361, 258–260. 10.1038/361258a0 8093806

[glia23620-bib-0006] Bechler, M. E. , Byrne, L. , & Ffrench‐Constant, C. (2015). CNS myelin sheath lengths are an intrinsic property of oligodendrocytes. Current Biology, 25(18), 2411–2416. 10.1016/j.cub.2015.07.056 26320951 PMC4580335

[glia23620-bib-0007] Bechler, M. E. , Swire, M. , & Ffrench‐Constant, C. (2017). Intrinsic and adaptive myelination ‐ a sequential mechanism for smart wiring in the brain. Developmental Neurobiology, 78, 68–79. 10.1002/dneu.22518 28834358 PMC5813148

[glia23620-bib-0009] Butt, A. , Colquhoun, K. , Tutton, M. , & Berry, M. (1994). Three‐dimensional morphology of astrocytes and oligodendrocytes in the intact mouse optic nerve. Journal of Neurocytology, 23(8), 469–485. 10.1007/BF01184071 7527074

[glia23620-bib-0010] Caramia, M. D. , Palmieri, M. G. , Desiato, M. T. , Boffa, L. , Galizia, P. , Rossini, P. M. , … Bernardi, G. (2004). Brain excitability changes in the relapsing and remitting phases of multiple sclerosis: A study with transcranial magnetic stimulation. Clinical Neurophysiology, 115(4), 956–965. 15003779 10.1016/j.clinph.2003.11.024

[glia23620-bib-0011] Castillo‐Padilla, D. , & Funke, K. (2016). Effects of chronic iTBS‐rTMS and enrighted environment on visual cortex early critical period and visual pattern discrimination in dark‐reared rats. Developmental Neurobiology, 76(1), 19–33. 10.1002/dneu.22296 25892203

[glia23620-bib-0012] Cavaliere, F. , Benito‐Munoz, M. , Panicker, M. , & Matute, C. (2013). NMDA modulates oligodenrocyte differentiation of subventricular zone cells through PKC activation. Frontiers in Cellular Neuroscience, 7, 261 10.3389/fncel.2013.00261 24391542 PMC3866621

[glia23620-bib-0013] Chong, S. C. , Rosenberg, S. S. , Fancy, S. P. , Zhao, C. , Shen, Y.‐A. A. , Hahn, A. T. , … Zhang, L. I. (2012). Neurite outgrowth inhibitor Nogo‐A establishes spatial segregation and extent of oligodendrocyte myelination. Proceedings of the National Academy of Sciences, 109(4), 1299–1304. 10.1073/pnas.1113540109 PMC3268264 22160722

[glia23620-bib-0015] Clarke, L. E. , Young, K. M. , Hamilton, N. B. , Li, H. , Richardson, W. D. , & Attwell, D. (2012). Properties and fate of oligodendrocyte progenitor cells in the corpus callosum, motor cortex, and piriform cortex of the mouse. The Journal of Neuroscience, 32(24), 8173–8185. 10.1523/JNEUROSCI.0928-12.2012 22699898 PMC3378033

[glia23620-bib-0016] Cohen, J. E. , & Fields, R. D. (2008). Activity‐dependent neuron–glial signaling by ATP and leukemia‐inhibitory factor promotes hippocampal glial cell development. Neuron Glia Biology, 4(1), 43–55. 10.1017/S1740925X09000076 19267953 PMC2756042

[glia23620-bib-0017] Croarkin, P. E. , Nakonezny, P. A. , Wall, C. A. , Murphy, L. L. , Sampson, S. M. , Frye, M. A. , & Port, J. D. (2016). Transcranial magnetic stimulation potentiates glutamatergic neurotransmission in depressed adolescents. Psychiatry Research: Neuroimaging, 247, 25–33. 10.1016/j.pscychresns.2015.11.005 26651598 PMC4716879

[glia23620-bib-0018] Cullen, C. L. , & Young, K. M. (2016). How does transcranial magnetic stimulation influence glial cells in the central nervous system? Frontiers in neural circuits, 10, 26 10.3389/fncir.2016.00026 27092058 PMC4820444

[glia23620-bib-0019] Dawson, M. R. L. , Polito, A. , Levine, J. M. , & Reynolds, R. (2003). NG2‐expressing glial progenitor cells: An abundant and widespread population of cycling cells in the adult rat CNS. Molecular and Cellular Neuroscience, 24(2), 476–488. 10.1016/S1044-7431(03)00210-0 14572468

[glia23620-bib-0020] Deng, Z.‐D. , Lisanby, S. H. , & Peterchev, A. V. (2013). Electric field depth–focality tradeoff in transcranial magnetic stimulation: Simulation comparison of 50 coil designs. Brain Stimulation, 6(1), 1–13. 10.1016/j.brs.2012.02.005 22483681 PMC3568257

[glia23620-bib-0021] Fang, Z.‐Y. , Li, Z. , Xiong, L. , Huang, J. , & Huang, X.‐L. (2010). Magnetic stimulation influences injury‐induced migration of white matter astrocytes. Electromagnetic Biology and Medicine, 29(3), 113–121. 20707645 10.3109/15368378.2010.500568

[glia23620-bib-0022] Ferrer, I. , Bernet, E. , Soriano, E. , Del Rio, T. , & Fonseca, M. (1990). Naturally occurring cell death in the cerebral cortex of the rat and removal of dead cells by transitory phagocytes. Neuroscience, 39(2), 451–458. 10.1016/0306-4522(90)90281-8 2087266

[glia23620-bib-0023] Franklin, K. B. , & Paxinos, G. (2007). The mouse brain in stereotaxic coordinates (3rd ed.). Cambridge, MA: Academic Press.

[glia23620-bib-0024] Franklin, R. , & Ffrench‐Constant, C. (2008). Remyelination in the CNS: From biology to therapy. Nature Reviews. Neuroscience, 9(11), 839–855. 10.1038/nrn2480 18931697

[glia23620-bib-0025] Gaede, G. , Tiede, M. , Lorenz, I. , Brandt, A. U. , Pfueller, C. , Dorr, J. , … Paul, F. (2018). Safety and preliminary efficacy of deep transcranial magnetic stimulation in MS‐related fatigue. Neurology: Neuroimmunology & Neuroinflammation, 5(1), e423 10.1212/nxi.0000000000000423 29259998 PMC5730816

[glia23620-bib-0026] Gard, A. , Burrell, M. , Pfeiffer, S. , Rudge, J. , & Williams, W. (1995). Astroglial control of oligodendrocyte survival mediated by PDGF and leukemia inhibitory factor‐like protein. Development, 121(7), 2187–2197. 7635062 10.1242/dev.121.7.2187

[glia23620-bib-0027] Gautier, H. O. , Evans, K. A. , Volbracht, K. , James, R. , Sitnikov, S. , Lundgaard, I. , … Franklin, R. J. (2015). Neuronal activity regulates remyelination via glutamate signalling to oligodendrocyte progenitors. Nature Communications, 6, 8518 10.1038/ncomms9518 PMC4600759 26439639

[glia23620-bib-0028] Gibson, E. M. , Purger, D. , Mount, C. W. , Goldstein, A. K. , Lin, G. L. , Wood, L. S. , … Monje, M. (2014). Neuronal activity promotes oligodendrogenesis and adaptive myelination in the mammalian brain. Science, 344(6183), 1252304 10.1126/science.1252304 24727982 PMC4096908

[glia23620-bib-0029] Grehl, S. , Viola, H. M. , Fuller‐Carter, P. I. , Carter, K. W. , Dunlop, S. A. , Hool, L. C. , … Rodger, J. (2015). Cellular and molecular changes to cortical neurons following low intensity repetitive magnetic stimulation at different frequencies. Brain Stimulation, 8(1), 114–123. 10.1016/j.brs.2014.09.012 25444593

[glia23620-bib-0030] Hallett, M. (2007). Transcranial magnetic stimulation: a primer. Neuron, 55(2), 187–199. 10.1016/j.neuron.2007.06.026 17640522

[glia23620-bib-0031] Hamilton, N. B. , Clarke, L. E. , Arancibia‐Carcamo, I. L. , Kougioumtzidou, E. , Matthey, M. , Káradóttir, R. , … Attwell, D. (2017). Endogenous GABA controls oligodendrocyte lineage cell number, myelination, and CNS internode length. Glia, 65(2), 309–321. 10.1002/glia.23093 27796063 PMC5214060

[glia23620-bib-0032] Hill, R. A. , Li, A. M. , & Grutzendler, J. (2018). Lifelong cortical myelin plasticity and age‐related degeneration in the live mammalian brain. Nature Neuroscience, 21(5), 683–695. 10.1038/s41593-018-0120-6 29556031 PMC5920745

[glia23620-bib-0033] Hill, R. A. , Patel, K. D. , Goncalves, C. M. , Grutzendler, J. , & Nishiyama, A. (2014). Modulation of oligodendrocyte generation during a critical temporal window after NG2 cell division. Nature Neuroscience, 17(11), 1518–1527. 10.1038/nn.3815 25262495 PMC4275302

[glia23620-bib-0034] Hines, J. H. , Ravanelli, A. M. , Schwindt, R. , Scott, E. K. , & Appel, B. (2015). Neuronal activity biases axon selection for myelination in vivo. Nature Neuroscience, 18(5), 683–689. 10.1038/nn.3992 25849987 PMC4414883

[glia23620-bib-0035] Hippenmeyer, S. , Vrieseling, E. , Sigrist, M. , Portmann, T. , Laengle, C. , Ladle, D. R. , & Arber, S. (2005). A developmental switch in the response of DRG neurons to ETS transcription factor signaling. PLoS Biology, 3(5), e159 10.1371/journal.pbio.0030159 15836427 PMC1084331

[glia23620-bib-0036] Hoogendam, J. , Ramakers, G. , & Di Lazzaro, V. (2010). Physiology of repetitive trancranial magnetic stimulation of the human brain. Brain Stimulation, 3(2), 95–118. 10.1016/j.brs.2009.10.005 20633438

[glia23620-bib-0037] Hoppenrath, K. , & Funke, K. (2013). Time‐course of changes in neuronal activity markers following iTBS‐TMS of the rat neocortex. Neuroscience Letters, 536, 19–23. 10.1016/j.neulet.2013.01.003 23328445

[glia23620-bib-0038] Hoppenrath, K. , Hartig, W. , & Funke, K. (2016). Intermittent theta‐burst transcranial magnetic stimulaiton alters electrical properties of fast‐spiking neocortical interneurons in an age‐dependent fashion. Frontiers in Neural Circuits, 10, 22 10.3389/fncir.2016.00022 27065812 PMC4811908

[glia23620-bib-0039] Hughes, E. , Kang, S. H. , Masahiro, F. , & Bergles, D. E. (2013). Oligodendrocyte progenitors balance growth with self repulsion to achieve homeostasis in the adult brain. Nature Neuroscience, 16(6), 668–676. 10.1038/nn.3390 23624515 PMC3807738

[glia23620-bib-0040] Hughes, E. G. , Orthmann‐Murphy, J. L. , Langseth, A. J. , & Bergles, D. E. (2018). Myelin remodeling through experience‐dependent oligodendrogenesis in the adult somatosensory cortex. Nature Neuroscience, 21(5), 696–706. 10.1038/s41593-018-0121-5 29556025 PMC5920726

[glia23620-bib-0041] Hulst, H. E. , Goldschmidt, T. , Nitsche, M. A. , de Wit, S. J. , van den Heuvel, O. A. , Barkhof, F. , … Geurts, J. J. G. (2017). rTMS affects working memory performance, brain activation and functional connectivity in patients with multiple sclerosis. Journal of Neurology, Neurosurgery & Psychiatry, 88(5), 386–394. 10.1136/jnnp-2016-314224 27974394

[glia23620-bib-0042] Ishibashi, T. , Dakin, K. A. , Stevens, B. , Lee, P. R. , Kozlov, S. V. , Stewart, C. L. , & Fields, R. D. (2006). Astrocytes promote myelination in response to electrical impulses. Neuron, 49(6), 823–832. 10.1016/j.neuron.2006.02.006 16543131 PMC1474838

[glia23620-bib-0043] Koudelka, S. , Voas, M. , Almeida, R. , Baraban, M. , Soetaert, J. , Meyer, M. , … Lyons, D. A. (2016). Individual neuronal subtypes exhibit diversity in CNS myelination mediated by synaptic vesicle release. Current Biology, 26, 1447–1455. 10.1016/j.cub.2016.03.070 27161502 PMC4906267

[glia23620-bib-0044] Kougioumtzidou, E. , Shimizu, T. , Hamilton, N. B. , Tohyama, K. , Sprengel, R. , Monyer, H. , … Richardson, W. D. (2017). Signalling through AMPA receptors on oligodendrocyte precursors promotes myelination by enhancing oligodendrocyte survival. eLife, 6, e28080 10.7554/eLife.28080 28608780 PMC5484614

[glia23620-bib-0045] Krasnow, A. M. , Ford, M. C. , Valdivia, L. E. , Wilson, S. W. , & Attwell, D. (2018). Regulation of developing myelin sheath elongation by oligodendrocyte calcium transients in vivo. Nature Neuroscience, 21(1), 24–28. 10.1038/s41593-017-0031-y 29230052 PMC6478117

[glia23620-bib-0091] Labedi, A. , Benali, A. , Mix, A. , Neubacher, U. , & Funke, K. (2014). Modulation of Inhibitory Activity Markers by Intermittent Theta‐burst Stimulation in Rat Cortex is NMDA‐receptor Dependent. Brain Stimulation, 7(3), 394–400. 10.1016/j.brs.2014.02.010 24656783

[glia23620-bib-0046] Lenz, M. , Galanis, C. , Müller‐Dahlhaus, F. , Opitz, A. , Wierenga, C. J. , Szabó, G. , … Vlachos, A. (2016). Repetitive magnetic stimulation induces plasticity of inhibitory synapses. Nature Communications, 7, 10020 10.1038/ncomms10020 PMC4729863 26743822

[glia23620-bib-0047] Lenz, M. , Platschek, S. , Priesemann, V. , Becker, D. , Willems, L. M. , Ziemann, U. , … Vlachos, A. (2015). Repetitive magnetic stimulation induces plasticity of excitatory postsynapses on proximal dendrites of cultured mouse CA1 pyramidal neurons. Brain Structure and Function, 220(6), 3323–3337. 10.1007/s00429-014-0859-9 25108309

[glia23620-bib-0048] Li, Q. , Brus‐Ramer, M. , Martin, J. H. , & McDonald, J. W. (2010). Electrical stimulation of the medullary pyramid promotes proliferation and differentiation of oligodendrocyte progenitor cells in the corticospinal tract of the adult rat. Neuroscience Letters, 479(2), 128–133. 10.1016/j.neulet.2010.05.043 20493923 PMC2922017

[glia23620-bib-0049] Makinodan, M. , Rosen, K. , Ito, S. , & Corfas, G. (2012). A critical period for social experience‐dependent oligodendrocyte maturation and myelination. Science, 337, 1357–1360. 10.1126/science.1220845 22984073 PMC4165613

[glia23620-bib-0050] Makowiecki, K. , Harvey, A. R. , Sherrard, R. M. , & Rodger, J. (2014). Low‐intensity repetitive transcranial magnetic stimulation improves abnormal visual cortical circuit topography and upregulates BDNF in mice. The Journal of Neuroscience, 34(32), 10780–10792. 10.1523/JNEUROSCI.0723-14.2014 25100609 PMC4122806

[glia23620-bib-0051] Malone, M. , Gary, D. , Yang, I. H. , Miglioretti, A. , Houdayer, T. , Thakor, N. , & McDonald, J. (2013). Neuronal activity promotes myelination via a cAMP pathway. Glia, 61(6), 843–854. 10.1002/glia.22476 23554117

[glia23620-bib-0053] Medina‐Fernández, F. J. , Luque, E. , Aguilar‐Luque, M. , Agüera, E. , Feijóo, M. , García‐Maceira, F. I. , … Túnez, I. (2017). Transcranial magnetic stimulation modifies astrocytosis, cell density and lipopolysaccharide levels in experimental autoimmune encephalomyelitis. Life Sciences, 169, 20–26. 27876534 10.1016/j.lfs.2016.11.011

[glia23620-bib-0054] Mensch, S. , Baraban, M. , Almeida, R. , Czopka, T. , Ausborn, J. , El Manira, A. , & Lyons, D. A. (2015). Synaptic vesicle release regulates myelin sheath number of individual oligodendrocytes in vivo. Nature Neuroscience, 18(5), 628–630. 10.1038/nn.3991 25849985 PMC4427868

[glia23620-bib-0055] Mori, F. , Codecà, C. , Kusayanagi, H. , Monteleone, F. , Boffa, L. , Rimano, A. , … Centonze, D. (2010). Effects of intermittent theta burst stimulation on spasticity in patients with multiple sclerosis. European Journal of Neurology, 17(2), 295–300. 10.1111/j.1468-1331.2009.02806.x 19863647

[glia23620-bib-0056] Mori, F. , Ljoka, C. , Magni, E. , Codecà, C. , Kusayanagi, H. , Monteleone, F. , … Centonze, D. (2011). Transcranial magnetic stimulation primes the effects of exercise therapy in multiple sclerosis. Journal of Neurology, 258(7), 1281–1287. 10.1007/s00415-011-5924-1 21286740

[glia23620-bib-0057] Mount, C. W. , & Monje, M. (2017). Wrapped to adapt: Experience‐dependent myelination. Neuron, 95, 743–755. 10.1016/j.neuron.2017.07.009 28817797 PMC5667660

[glia23620-bib-0058] Müller‐Dahlhaus, F. , & Vlachos, A. (2013). Unraveling the cellular and molecular mechanisms of repetitive magnetic stimulation. Frontiers in molecular neuroscience, 6, 50 10.3389/fnmol.2013.00050 24381540 PMC3865432

[glia23620-bib-0059] Müller, M. B. , Toschi, N. , Kresse, A. E. , Post, A. , & Keck, M. E. (2000). Long‐term repetitive transcranial magnetic stimulation increases the expression of brain‐derived neurotrophic factor and cholecystokinin mRNA, but not neuropeptide tyrosine mRNA in specific areas of rat brain. Neuropsychopharmacology, 23(2), 205–215. 10.1016/S0893-133X(00)00099-3 10882847

[glia23620-bib-0060] Murtie, J. C. , Macklin, W. B. , & Corfas, G. (2007). Morphometric analysis of oligodendrocytes in the adult mouse frontal cortex. Journal of Neuroscience Research, 85(10), 2080–2086. 10.1002/jnr.21339 17492793

[glia23620-bib-0061] Nagy, B. , Hovhannisyan, A. , Barzan, R. , Chen, T.‐J. , & Kukley, M. (2017). Different patterns of neuronal activity trigger distinct responses of oligodendrocyte precursor cells in the corpus callosum. PLoS Biology, 15(8), e2001993 10.1371/journal.pbio.2001993 28829781 PMC5567905

[glia23620-bib-0062] Nielsen, J. F. , Sinkjaer, T. , & Jakobsen, J. (1996). Treatment of spasticity with repetitive magnetic stimulation; a double‐blind placebo‐controlled study. Multiple Sclerosis, 2(5), 227–232. 10.1177/135245859600200503 9050361

[glia23620-bib-0063] O'Rourke, M. , Cullen, C. L. , Auderset, L. , Pitman, K. A. , Achatz, D. , Gasperini, R. , & Young, K. M. (2016). Evaluating tissue‐specific recombination in a Pdgfrα‐CreERT2 transgenic mouse line. PLoS One, 11(9), e0162858. 10.1371/journal.pone.0162858 PMC5023134 27626928

[glia23620-bib-0064] Osanai, Y. , Shimizu, T. , Mori, T. , Yoshimura, Y. , Hatanaka, N. , Nambu, A. , … Ikenaka, K. (2017). Rabies virus‐mediated oligodendrocyte labeling reveals a single oligodendrocyte myelinates axons from distinct brain regions. Glia, 65(1), 93–105. 10.1002/glia.23076 27759175

[glia23620-bib-0065] Palser, A. , Norman, A. , Saffell, J. , & Reynolds, R. (2009). Neural cell adhesion molecule stimulates survival of premyelinating oligodendrocytes via the fibroblast growth factor receptor. Journal of Neuroscience Research, 87, 3356–3368. 10.1002/jnr.22248 19739251

[glia23620-bib-0066] Payne, S. C. , Bartlett, C. A. , Savigni, D. L. , Harvey, A. R. , Dunlop, S. A. , & Fitzgerald, M. (2013). Early proliferation does not prevent the loss of oligodendrocyte progenitor cells during the chronic phase of secondary degeneration in a CNS white matter tract. PLoS One, 8(6), e65710 10.1371/journal.pone.0065710 23776532 PMC3679191

[glia23620-bib-0067] Pepper, R. E. , Pitman, K. A. , Cullen, C. L. , & Young, K. M. (2018). How do cells of the Oligodendrocyte lineage affect neuronal circuits to influence motor function, memory and mood? Frontiers in Cellular Neuroscience, 12(399), 12 10.3389/fncel.2018.00399 30524235 PMC6262292

[glia23620-bib-0068] Pitman, K. A. , & Young, K. (2016). Activity‐dependent calcium signalling in oligodendrocyte generation. The International Journal of Biochemistry and Cell Biology, 77, 30–34. 10.1016/j.biocel.2016.05.018 27233230

[glia23620-bib-0069] Rivers, L. E. , Young, K. M. , Rizzi, M. , Jamen, F. , Psachoulia, K. , Wade, A. , … Richardson, W. D. (2008). PDGFRA/NG2 glia generate myelinating oligodendrocytes and piriform projection neurons in adult mice. Nature Neuroscience, 11(12), 1392–1401. 10.1038/nn.2220 18849983 PMC3842596

[glia23620-bib-0070] Rossi, S. , Hallett, M. , Rossini, P. M. , & Pascual‐Leone, A. (2009). Safety, ethical considerations, and application guidelines for the use of transcranial magnetic stimulation in clinical practice and research. Clinical Neurophysiology, 120(12), 2008–2039. 10.1016/j.clinph.2009.08.016 19833552 PMC3260536

[glia23620-bib-0071] Sherafat, M. A. , Heibatollahi, M. , Mongabadi, S. , Moradi, F. , Javan, M. , & Ahmadiani, A. (2012). Electromagnetic field stimulation potentiates endogenous myelin repair by recruiting subventricular neural stem cells in an experimental model of white matter demyelination. Journal of Molecular Neuroscience, 48(1), 144–153. 22588976 10.1007/s12031-012-9791-8

[glia23620-bib-0072] Srinivas, S. , Watanabe, T. , Lin, C.‐S. , William, C. M. , Tanabe, Y. , Jessell, T. M. , & Costantini, F. (2001). Cre reporter strains produced by targeted insertion of EYFP and ECFP into the ROSA26 locus. BMC Developmental Biology, 1(1), 4 10.1186/1471-213X-1-4 11299042 PMC31338

[glia23620-bib-0073] Sun, L. O. , Mulinyawe, S. B. , Collins, H. Y. , Ibrahim, A. , Li, Q. , Simon, D. J. , … Barres, B. A. (2018). Spatiotemporal control of CNS myelination by oligodendrocyte programmed cell death through the TFEB‐PUMA axis. Cell, 175(7), 1811–1826.e1821. 10.1016/j.cell.2018.10.044 30503207 PMC6295215

[glia23620-bib-0074] Tang, A. , Thickbroom, G. , & Rodger, J. (2017). Repetitive transcranial magnetic stimulation of the brain: Mechanisms from animal and experimental models. The Neuroscientist, 23(1), 82–94. 10.1177/1073858415618897 26643579

[glia23620-bib-0075] Tang, A. D. , Hong, I. , Boddington, L. J. , Garrett, A. R. , Etherington, S. , Reynolds, J. N. , & Rodger, J. (2016). Low‐intensity repetitive magnetic stimulation lowers action potential threshold and increases spike firing in layer 5 pyramidal neurons in vitro. Neuroscience, 335, 64–71. 10.1016/j.neuroscience.2016.08.030 27568058

[glia23620-bib-0076] Tang, A. D. , Lowe, A. S. , Garrett, A. R. , Woodward, R. , Bennett, W. , Canty, A. J. , … Gersner, R. (2016). Construction and Evaluation of Rodent‐Specific rTMS Coils. Frontiers in neural circuits, 10, 47 10.3389/fncir.2016.00047 27445702 PMC4928644

[glia23620-bib-0077] Thomaidou, D. , Mione, C. M. , Cavanagh, J. F. R. , & Parnavelas, J. G. (1997). Apoptosis and its relation to the cell cycle in the developing cerebral cortex. Journal of Neuroscience, 17(3), 1075–1085. 10.1523/JNEUROSCI.17-03-01075.1997 8994062 PMC6573180

[glia23620-bib-0078] Trapp, B. D. , Nishiyama, A. , Cheng, D. , & Macklin, W. B. (1997). Differentiation and death of premyelinating oligodendrocytes in developing rodent brain. Journal of Cell Biology, 137(2), 459–468. 10.1083/jcb.137.2.459 9128255 PMC2139778

[glia23620-bib-0079] Tripathi, R. , Jackiewicz, M. , McKenzie, I. , Kougioumtzidou, E. , Grist, M. , & Richardson, W. D. (2017). Remarkable stability of myelinating oligodendrocytes in mice. Cell Reports, 21, 316–323. 10.1016/j.celrep.2017.09.050 29020619 PMC5643547

[glia23620-bib-0080] Vlachos, A. , Müller‐Dahlhaus, F. , Rosskopp, J. , Lenz, M. , Ziemann, U. , & Deller, T. (2012). Repetitive magnetic stimulation induces functional and structural plasticity of excitatory postsynapses in mouse organotypic hippocampal slice cultures. The Journal of Neuroscience, 32(48), 17514–17523. 10.1523/JNEUROSCI.0409-12.2012 23197741 PMC6621866

[glia23620-bib-0081] Vucic, S. , Ziemann, U. , Eisen, A. , Hallett, M. , & Kiernan, M. C. (2013). Transcranial magnetic stimulation and amyotrophic lateral sclerosis: Pathophysiological insights. Journal of Neurology, Neurosurgery & Psychiatry, 84, 1161–1170. 10.1136/jnnp-2012-304019 PMC3786661 23264687

[glia23620-bib-0085] Wong, A. W. , Xiao, J. , Kemper, D. , Kilpatrick, T. J. , & Murray, S. S. (2013). Oligodendroglial expression of TrkB independently regulates myelination and progenitor cell proliferation. The Journal of Neuroscience, 33(11), 4947–4957. 10.1523/JNEUROSCI.3990-12.2013 23486965 PMC6619007

[glia23620-bib-0086] Xiao, L. , Ohayon, D. , McKenzie, I. A. , Sinclair‐Wilson, A. , Wright, J. L. , Fudge, A. D. , … Richardson, W. D. (2016). Rapid production of new oligodendrocytes is required in the earliest stages of motor skill learning. Nature Neuroscience, 19(9), 1210–1217. 27455109 10.1038/nn.4351 PMC5008443

[glia23620-bib-0087] Xiao, J. , Wong, A. W. , Willingham, M. M. , van den Buuse, M. , Kilpatrick, T. J. , & Murray, S. S. (2010). Brain‐derived neurotrophic factor promotes central nervous system myelination via a direct effect upon oligodendrocytes. Neurosignals, 18(3), 186–202. 10.1159/000323170 21242670

[glia23620-bib-0088] Young, K. M. , Psachoulia, K. , Tripathi, R. B. , Dunn, S.‐J. , Cossell, L. , Attwell, D. , … Richardson, W. D. (2013). Oligodendrocyte dynamics in the healthy adult CNS: Evidence for myelin remodeling. Neuron, 77(5), 873–885. 10.1016/j.neuron.2013.01.006 23473318 PMC3842597

[glia23620-bib-0089] Zhang, N. , Xing, M. , Wang, Y. , Tao, H. , & Cheng, Y. (2015). Repetitive transcranial magnetic stimulation enhances spatial learning and synaptic plasticity via the VEGF and BDNF–NMDAR pathways in a rat model of vascular dementia. Neuroscience, 311, 284–291. 10.1016/j.neuroscience.2015.10.038 26518460

