# Supplementary figures and images for: Low‐intensity transcranial magnetic stimulation promotes the survival and maturation of newborn oligodendrocytes in the adult mouse brain
Source: Glia. 2019 Apr 16;67(8):1462–77. doi: 10.1002/glia.23620 (PMC6790715; doi:10.1002/glia.23620)

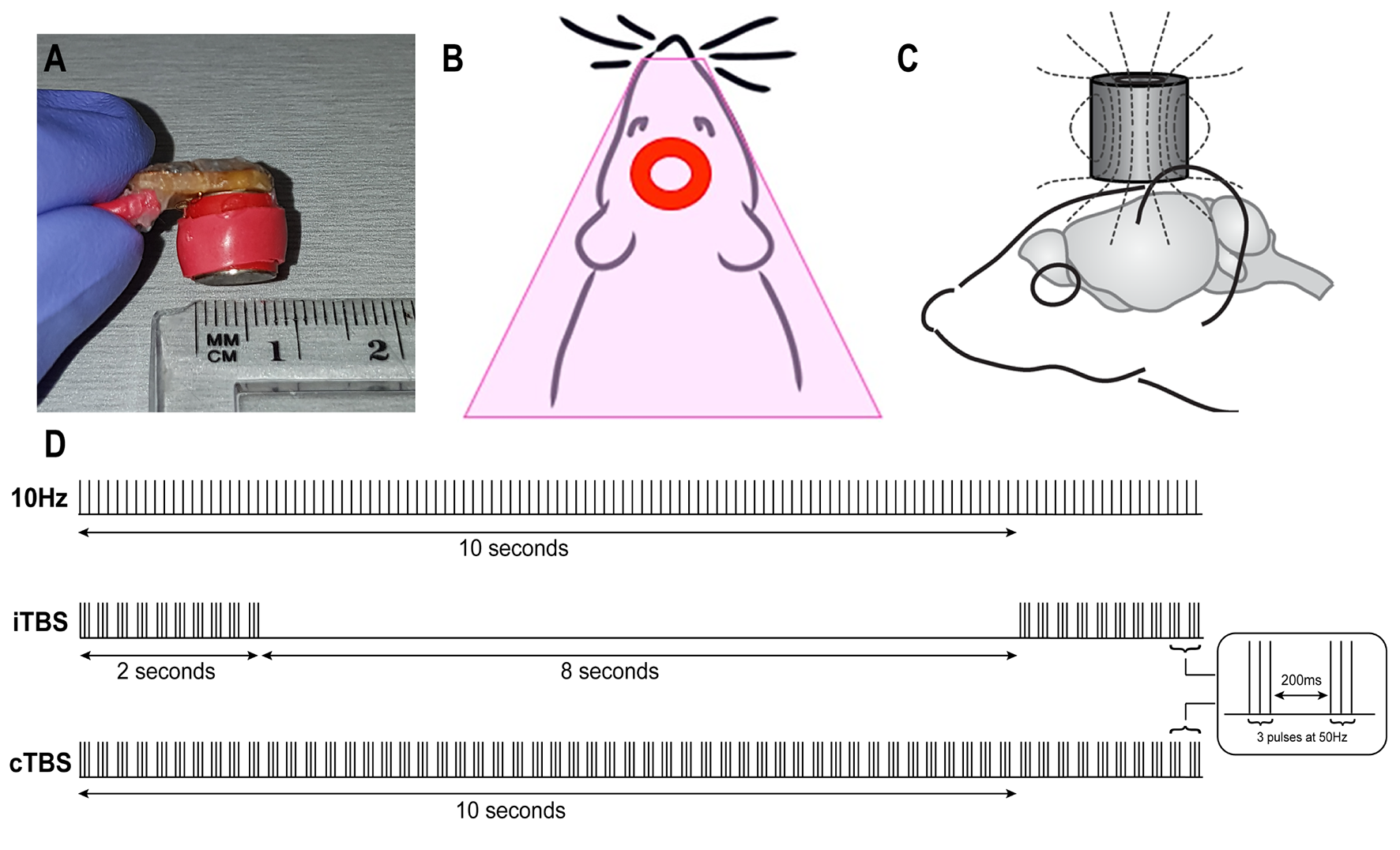

Supplement: Supplementary file 1 — Figure S1 Administration of LI‐rTMS to mice using a specialised rodent coil (A) Photograph of the 8 mm circular rodent coil used to deliver LI‐rTMS to mice in this study (Tang et al., 2016). (B) Schematic showing the method of restraint (plastic body contour shape restraint bag; pink), placement of the coil (red circle) and approximate region where the greatest current is generated (red circle). (C) Illustration of the magnetic flux generated by the coil. Image approximately to scale. (D) Illustration of the 10 Hz, intermittent theta burst (iTBS) and continuous theta burst (cTBS) stimulation patterns delivered in this study. Each bar represents a magnetic pulse that generates a reciprocal pulsed current in the underlying brain. Mice received a total of 600 pulses delivered in one of these patterns, or no pulses, i.e., sham stimulation, where the coil was not activated. This treatment was repeated daily for up to 28 days. [file GLIA-67-1462-s001.tif]

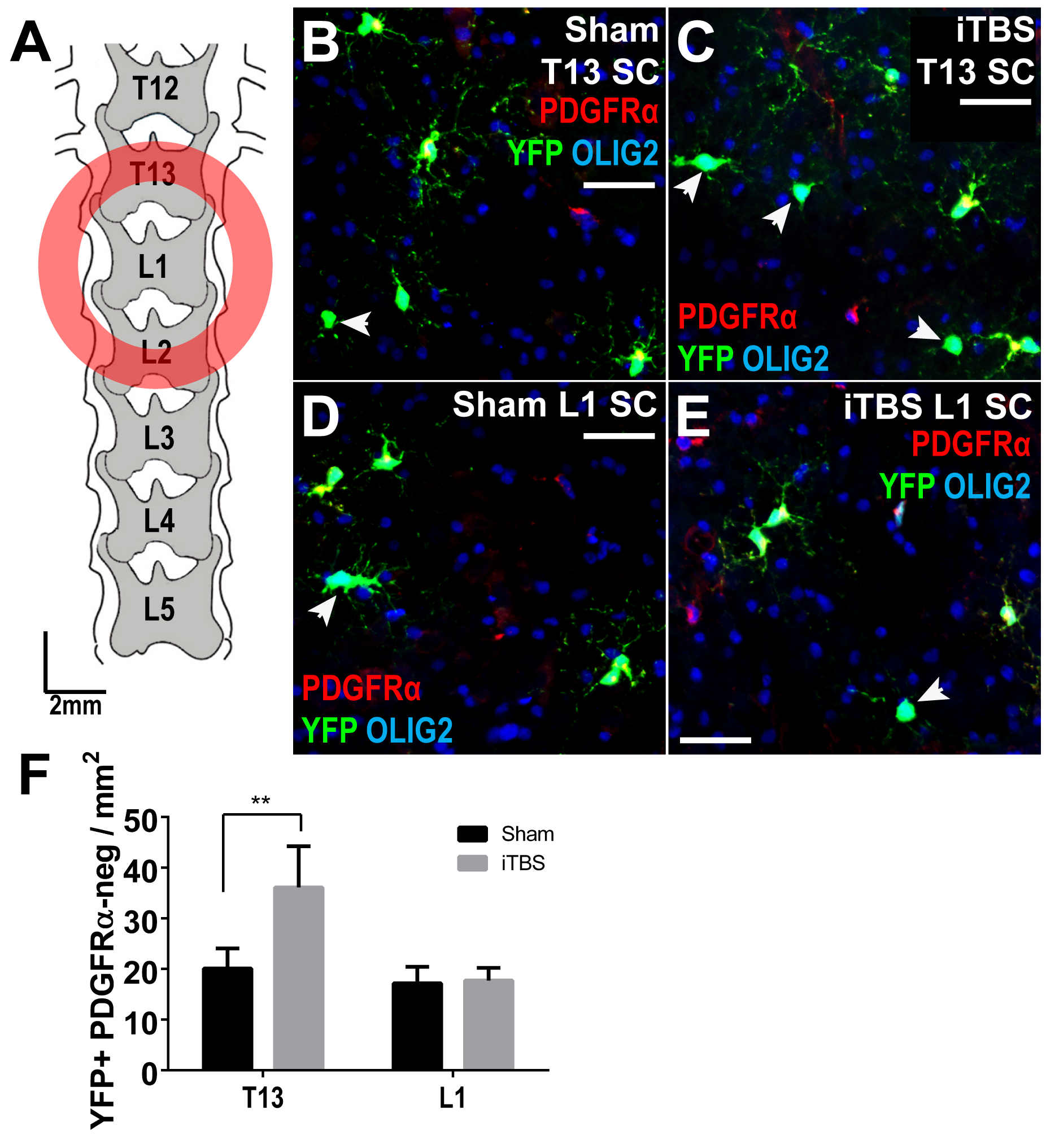

Supplement: Supplementary file 2 — Figure S2 iTBS increases the number of new oligodendrocytes in the adult mouse spinal cord (A) P83 Pdgfrα‐CreER T2 ::Rosa26YFP transgenic mice received 300 mg/kg Tamoxifen for four consecutive days. At P90, mice were randomly assigned to receive either sham or iTBS stimulation of the spinal cord, daily for 14 consecutive days. For spinal cord stimulation, the coil (red circle) was held over the spinal cord so that the front of the coil was positioned over the T13 vertebrae. Mice were perfusion fixed and the spinal cord collected for immunohistochemical analysis 1 day after treatment cessation. (B‐E) Low magnification confocal images of transverse spinal cord cryosections (30 μm) collected at the level of the T13 (B‐C; underneath the circumference of the coil) or L1 (D‐E; underneath the center of the coil) vertebrae of sham and iTBS mice. Sections were immunolabeled to detect PDGFRα (red), YFP (green) and OLIG2 (blue). (F) Graphical representation of YFP+, PDGFRα‐negative, OLIG2+ new oligodendrocyte number in T13 and L1 spinal cord 1 day after 14 days of sham (black) or iTBS (grey) treatment. iTBS significantly increased new oligodendrocyte number in the T13 spinal cord but had no effect on new oligodendrocyte number in the L1 spinal cord [two‐way ANOVA treatment F (1,8) = 8.32 p = 0.02; spinal cord region F (1,8) = 13.79, p = 0.0059; interaction F (1,8) = 7.278, p = 0.027]. Asterisks denote statistical significance identified by Bonferroni post hoc analysis, **p < 0.01. [file GLIA-67-1462-s002.tif]

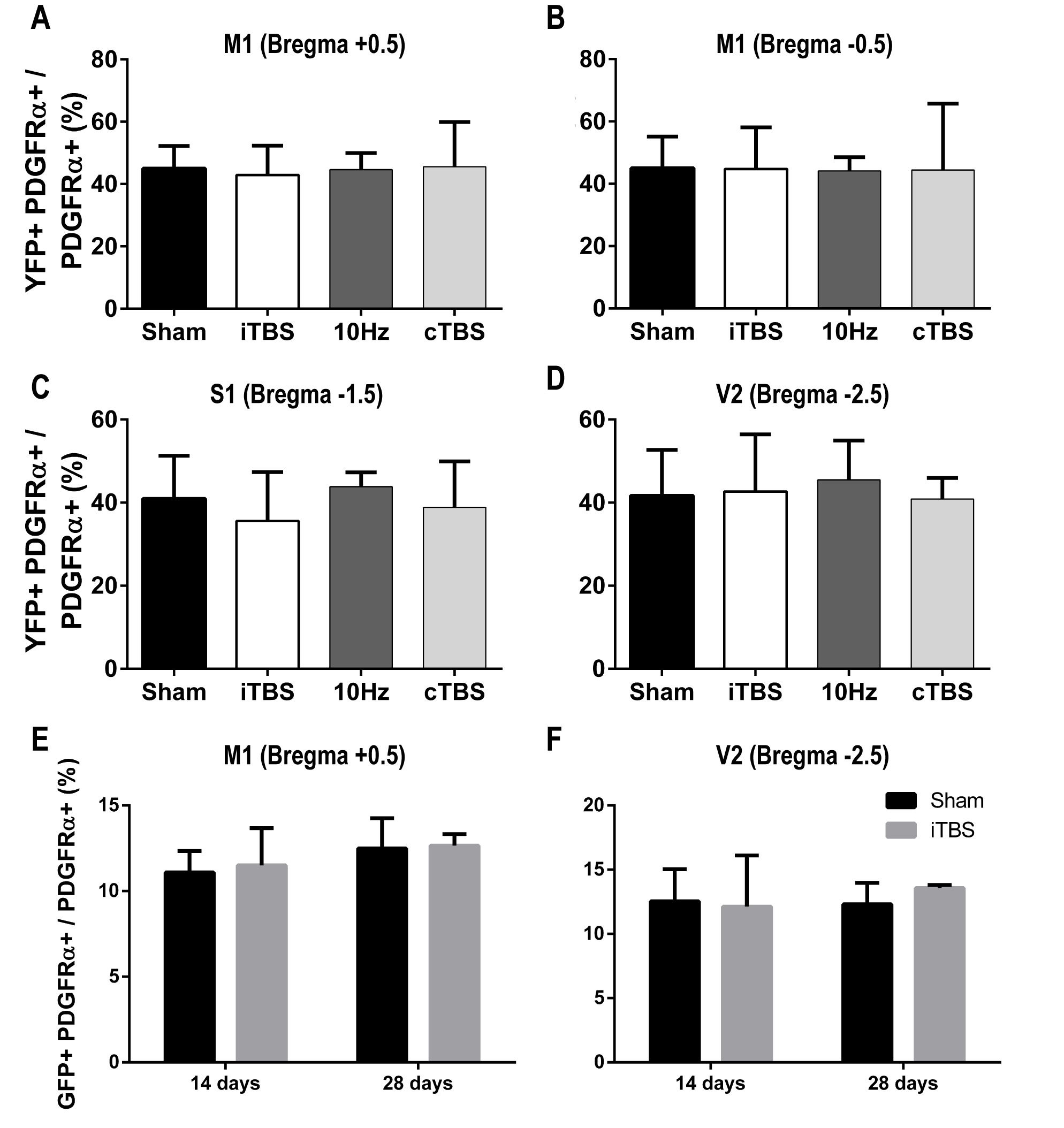

Supplement: Supplementary file 3 — Figure S3 An equivalent proportion of the OPC population became fluorescently labeled in sham and LI‐ rTMS‐treated mice (A‐D) P83 Pdgfrα‐CreER T2 ::Rosa26YFP transgenic mice received 300 mg/kg Tamoxifen for four consecutive days. At P90, mice were randomly assigned to a treatment group, receiving either sham stimulation, iTBS, 10 Hz or cTBS, daily for 14 consecutive days. Mice were perfusion fixed for immunohistochemical analysis 1 day after treatment cessation. The proportion of OPCs (PDGFRα+) that had undergone recombination to express yellow fluorescent protein (YFP) was quantified in the primary motor cortex (M1) at Bregma +0.5 [A: treatment F (3,16) = 0.05, p = 0.98] and Bregma −0.5 [B: treatment F (3,16) = 0.002, p = 0.99], the primary somatosensory cortex (S1) at Bregma −1.5 [C: treatment F (3,16) = 0.41, p = 0.74] and the secondary visual cortex (V2) at Bregma −2.5 [D: treatment F (3,16) = 0.13, p = 0.93] and is represented graphically. Data are expressed as mean + SD for n = 4 mice per treatment group. Data were analyzed by a 1‐way ANOVA. No significant difference was detected in the proportion of OPCs that recombined in any treatment group. (E‐F) P83 Pdgfrα‐CreER T2 ::Tau‐mGFP transgenic mice received 300 mg/kg Tamoxifen for four consecutive days. At P90, mice were randomly assigned to a treatment group, receiving either sham stimulation or iTBS for 14 or 28 consecutive days. Mice were perfusion fixed for immunohistochemical analysis 1 day after treatment cessation. The proportion of OPCs (PDGFRα+) that express green fluorescent protein (GFP) was quantified in M1 at Bregma +0.5 [E: treatment F (1,8) = 0.09, p = 0.76; treatment duration F (1,8) = 1.97, p = 0.19; interaction F (1,8) = 0.01, p = 0.89] and V2 at Bregma −2.5 [F: treatment F (1,8) = 0.01, p = 0.91, duration: F (1,8) = 0.18, p = 0.68, interaction: F (1,8) = 0.01, p = 0.90] and is graphically represented. Data are expressed as mean + SD for n = 3 mice per treatment group. Data were analyzed by a t [file GLIA-67-1462-s003.tif]

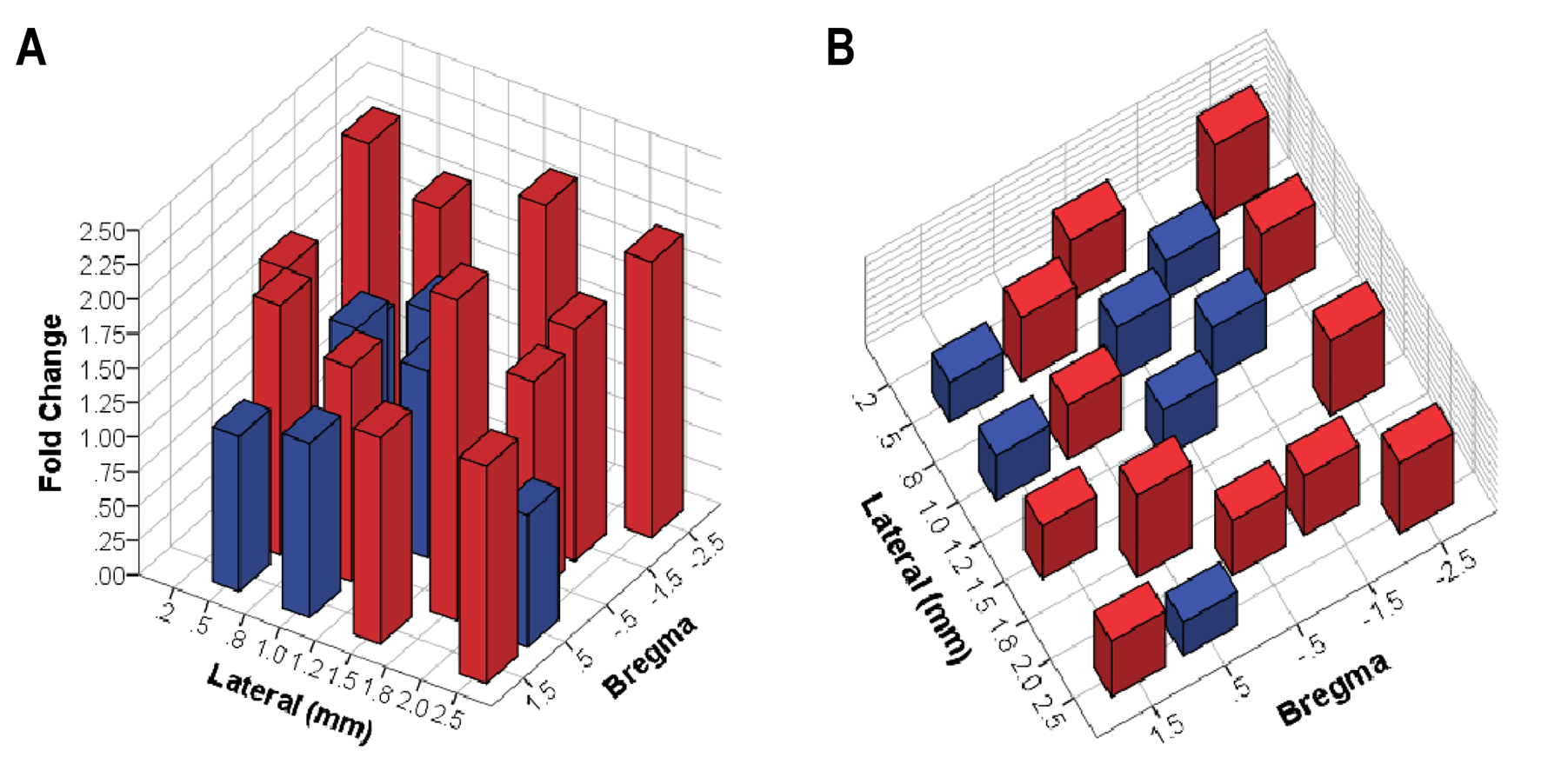

Supplement: Supplementary file 4 — Figure S4 Visual representation of new oligodendrocyte number in iTBS mice relative to sham stimulated controls at distinct Lateral and Bregma co‐ordinates (A) P83 Pdgfrα‐CreER T2 ::Rosa26YFP transgenic mice received 300 mg/kg Tamoxifen for 4 consecutive days. At P90, mice were randomly assigned to a treatment group, receiving either sham stimulation or iTBS, daily for 14 consecutive days. Mice were perfusion fixed for immunohistochemical analysis 1 day after treatment cessation. Coronal brain sections were collected from anatomically defined regions underneath the electromagnetic coil, and immunohistochemistry performed to quantify the density of YFP+ PDGFRα‐negative OLIG2+ new oligodendrocytes in each region. As the electromagnetic coil was circular, and there was no obvious difference between the right and left hemispheres, data for each hemisphere was combined to obtain a single density measure for newborn oligodendrocytes that could be mapped to a single anatomical point corresponding to a Bregma level (z‐axis) and lateral distance from the midline (x‐axis). To develop a visual representation of the effect of iTBS, the density of new oligodendrocytes detected in iTBS mice was divided by the density of new oligodendrocytes in sham stimulated mice, to obtain a relative fold change in oligodendrogenesis (y‐axis) at each anatomical point. Blue bars indicate regions of the brain where new oligodendrocyte density was equivalent between sham and iTBS treated mice (fold change of 0.95‐1.49). Red bars indicate brain regions, where the density of new oligodendrocytes in iTBS‐treated mice was ≥1.5 fold higher than sham‐stimulated mice. Fold change was calculated from the average data of n = 5 mice per treatment group provided in Table S1. (B) The graph depicted in (A) has been rotated to more clearly illustrate the location of brain regions in which iTBS had no effect on new oligodendrocyte density (blue) and the location of regions where iTBS increased new oligodendrocyt [file GLIA-67-1462-s004.tif]

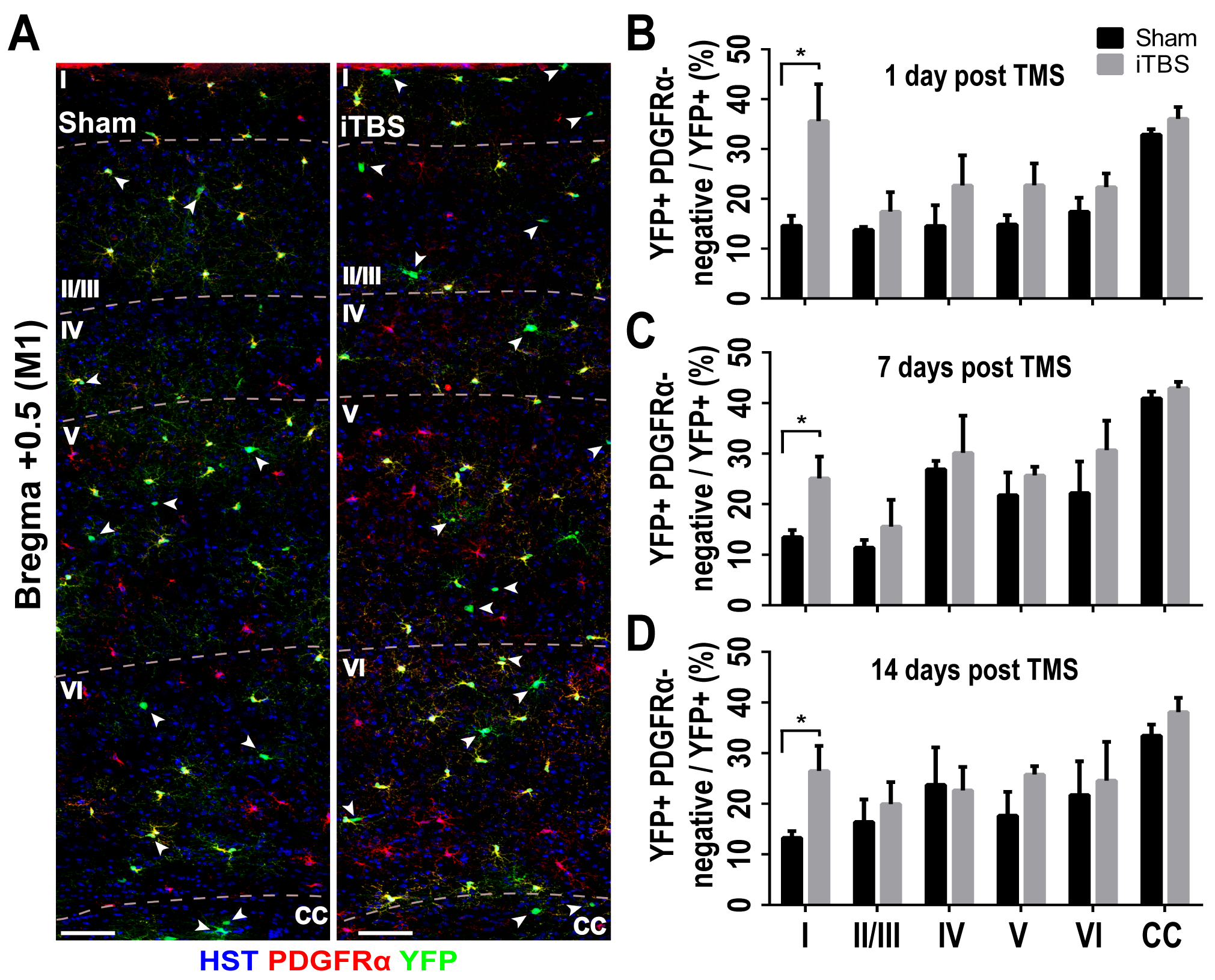

Supplement: Supplementary file 5 — Figure S5 iTBS does not increase new oligodendrocyte number in all M1 cortical layers (A) Low magnification confocal images of the primary motor cortex (M1) of Pdgfrα‐CreER T2 ::Rosa26‐YFP mice that were perfused 1 day after they received 14 days of sham stimulation (left) or iTBS (right), stained to detect PDGFRα (red), YFP (green) and Hoechst 33342 (HST; blue). (B‐D) Graph showing the proportion of YFP+ cells that are newly differentiated oligodendrocytes (PDGFRα‐negative, OLIG2+) in each layer of M1 and the CC of mice that received 14 days of sham or iTBS treatment and were perfusion fixed for analysis 1 later [B: n = 5 mice per treatment, two‐way ANOVA treatment F (1, 48) = 13.79, p = 0.0005; layer F (5, 48) = 6.44, p = 0.0001; interaction F (5, 48) = 1.532, p = 0.19], 7 days later [C: n = 4 mice per treatment, two‐way ANOVA treatment F (1, 36) = 5.40, p = 0.028; layer F (5, 36) = 10.79, p < 0.0001; interaction F (5, 36) = 0.39, p = 0.84] or 14 days later [D: n = 4 mice per treatment, two‐way ANOVA treatment F (1, 36) = 11.77, p = 0.0018; layer F (5, 36) = 11.26, p < 0.0001; interaction F (5, 36) = 1.73, p = 0.15]. Data are presented as mean + SD. Asterisks denote significant differences identified by Bonferroni post hoc analysis, *p < 0.05. Scale bars represent 40 μm. Arrowheads identify YFP+ OLIG2+ PDGFRα‐negative new oligodendrocytes. White dash lines identify the boundaries of each cortical layer (I‐VI) and the underlying corpus callosum (CC). [file GLIA-67-1462-s005.tif]

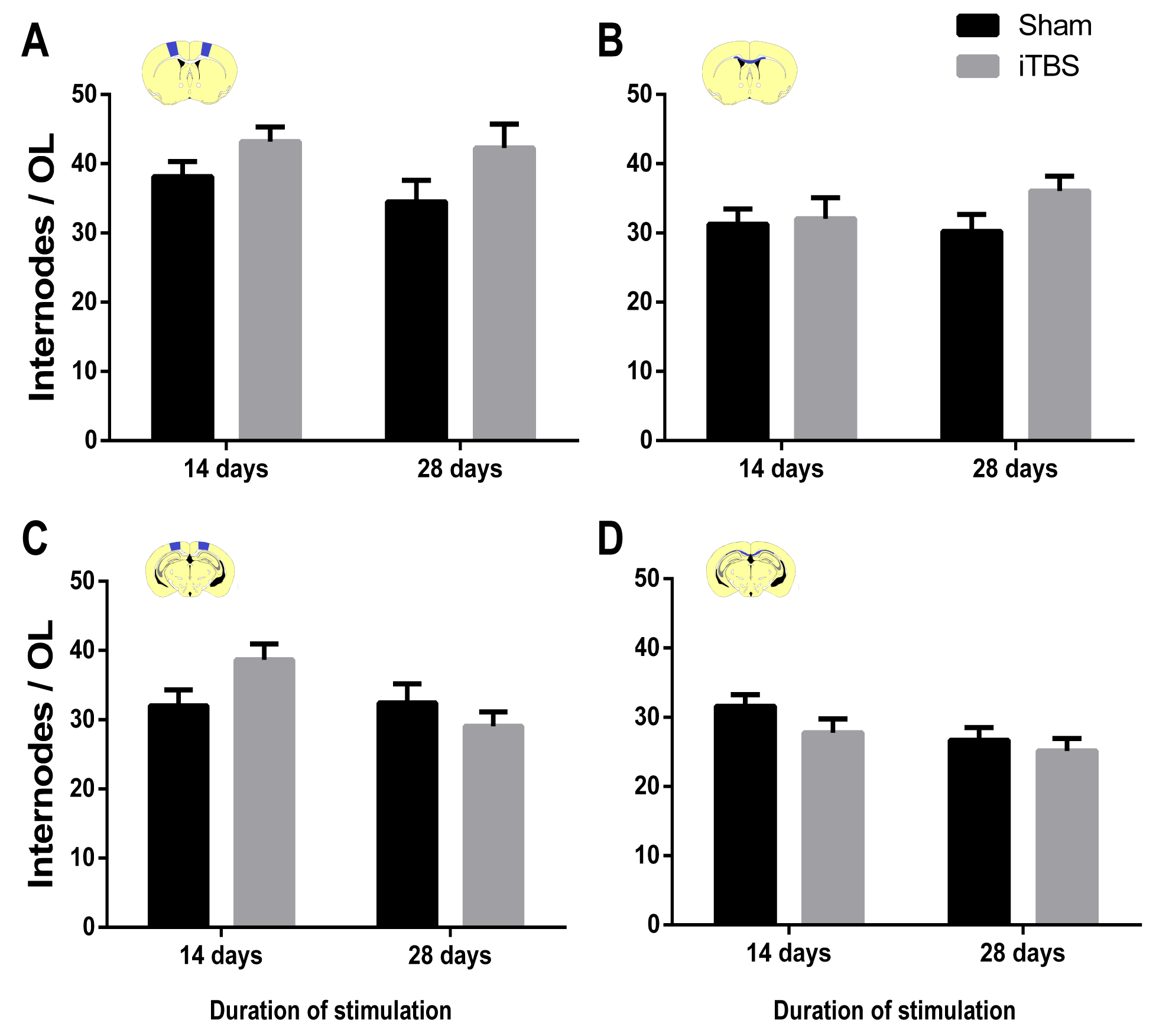

Supplement: Supplementary file 6 — Figure S6 iTBS does not influence the number of internodes elaborated by differentiating oligodendrocytes (A‐D) P83 Pdgfrα‐CreER T2 ::Tau‐mGFP transgenic mice received 300 mg/kg Tamoxifen for 4 consecutive days. At P90, mice were randomly assigned to a treatment group, receiving either sham stimulation or iTBS for 14 or 28 consecutive days. Mice were perfusion fixed for immunohistochemical analysis 1 day after treatment cessation. The number of internodes elaborated by individual GFP+ OLIG2+ new oligodendrocytes with myelinating morphology was quantified in the primary motor cortex (M1) [A: treatment F (1, 106) = 3.14, p = 0.08; treatment duration F (1, 106) = 0.73, p = 0.39; interaction F (1, 106) = 0.28, p = 0.59] and its underlying corpus callosum (C) [B: treatment F (1, 58) = 1.81, p = 0.18; treatment duration F (1, 58) = 0.36, p = 0.55; interaction F (1, 58) = 1.48, p = 0.31] or the secondary visual cortex (V2) [C: treatment F (1, 65) = 0.46, p = 0.49; treatment duration F (1, 65) = 3.61, p = 0.07; interaction F (1, 65) = 3.46, p = 0.08] and its underlying CC [D: treatment F (1, 50) = 1.70, p = 0.19; treatment duration F (1, 50) = 3.43, p = 0.07; interaction F (1, 50) = 0.31, p = 0.57]. Data are expressed as mean + SEM and were analyzed by two‐way ANOVA. Oligodendrocytes were imaged from n = 3 mice per treatment group. [file GLIA-67-1462-s006.tif]
