# Supplementary material for: Low‐intensity transcranial magnetic stimulation promotes the survival and maturation of newborn oligodendrocytes in the adult mouse brain
Source: Glia. 2019 Apr 16;67(8):1462–77. doi: 10.1002/glia.23620 (PMC6790715; doi:10.1002/glia.23620)
Supplement: Supplementary file 7 — Table S1 LI‐rTMS increases regional oligodendrocyte addition. [file GLIA-67-1462-s007.docx]

| **Bregma  co-ordinate** | **Region** | **Sham** mean OLs/mm^2^ (SD) | **iTBS** mean OLs/mm^2^ (SD) | **Main effects** (2-Way ANOVA) | **Bonferroni adjusted P value** |
| --- | --- | --- | --- | --- | --- |
| +1.5 | Cg1 | 26.72 (2.7) | 50.97 (10.9) | P_region_ = 0.53  P_trt_ = 0.0007*  P_int_ = 0.33 | 0.02* |
|  | M2 | 27.72 (1.9) | 35.09 (5.7) |  | >0.99 |
|  | M1 | 25.75 (2.6) | 46.66 (15.6) |  | 0.051 |
|  | Fr3 | 32.41 (4.7) | 42.40 (15.4) |  | 0.80 |
| +0.5 | Cg1 | 30.53 (3.4) | 55.16 (8.5) | P_region_ = 0.04*  P_trt_ < 0.0001*  P_int_ = 0.06 | 0.003* |
|  | M2 | 21.38 (6.2) | 36.48 (6.8) |  | 0.10 |
|  | M1 | 24.55 (4.5) | 45.65 (13.1) |  | 0.0014* |
|  | S1 | 33.47 (5.4) | 34.66 (4.2) |  | >0.99 |
| -0.5 | RSD | 38.56 (11.5) | 66.18 (13.8) | P_region_ = 0.07  P_trt_ = 0.002*  P_int_ = 0.78 | 0.09 |
|  | M2 | 44.30 (14.9) | 65.18 (23.3) |  | 0.29 |
|  | M1 | 36.36 (8.4) | 49.22 (8.1) |  | >0.99 |
|  | S1 | 27.20 (9.1) | 42.51 (11.1) |  | 0.72 |
| -1.5 | RSD | 38.97 (4.4) | 42.39 (12.4) | P_region_ = 0.36  P_trt_ = 0.051  P_int_ = 0.61 | >0.99 |
|  | PtA | 32.83 (12.1) | 46.51 (6.9) |  | 0.24 |
|  | S1 | 28.66 (4.5) | 38.63 (9.0) |  | 0.58 |
| -2.5 | RSGc | 29.05 (23.1) | 57.75 (15.23) | P_region_ = 0.31  P_trt_ = 0.0003*  P_int_ = 0.79 | 0.06 |
|  | RSD | 24.20 (5.8) | 43.84 (7.5) |  | 0.31 |
|  | V1 | 20.55 (4.0) | 38.07 (18.1) |  | 0.44 |
|  | V2 | 21.06 (1.7) | 50.32 (7.9) |  | 0.04* |
| **Table S1.** LI-rTMS increases regional oligodendrocyte addition.  Adult *Pdgfrα-CreER^T2^::Rosa26YFP* mice received tamoxifen from P83-P86. From P90, mice received either repetitive low intensity transcranial magnetic stimulation (LI-rTMS), as intermittent theta burst (iTBS) stimulation (n=3-5) or sham stimulation (n=3-4) for 14 consecutive days and were perfusion fixed 1 day later. Oligodendrocyte addition was quantified from immunohistochemistry detecting PDGFRα, YFP and OLIG2 (see **Figure 1-2**) in 30µm brain cryosections sampled from specific Bregma co-ordinates that spanned the region underneath the rodent TMS coil. The number of YFP^+^ OLIG2^+^ PDGFRα-negative new oligodendrocytes was quantified within defined cortical regions (Franklin and Paxinos, 2007) and expressed as the number of new oligodendrocytes (OLs) per mm^2^. iTBS significantly increases oligodendrocyte addition primarily within cortical regions located under the outer circumference of the coil, where the greatest current is generated (Tang et al., 2016b). *Cg1:* cingulate cortex area 1, *M2*: secondary motor cortex; *M1*: primary motor cortex, *Fr3:* frontal cortex area 3, *S1:* primary somatosensory cortex, *RSD:* retrosplenal dysgranular cortex, *PtA:* parietal association cortex, *RSGc:* retrosplenal granular cortex c region, *V1:* primary visual cortex, *V2:* secondary visual cortex. *denotes statistical significance (P<0.05). | | | | | |
